# Supplementary material for: Measuring social determinants of health in the All of Us Research Program
Source: Sci Rep. 2024 Apr 16;14:8815. doi: 10.1038/s41598-024-57410-6 (PMC11021514; doi:10.1038/s41598-024-57410-6)
Supplement: Supplementary file 1 — Supplementary Information. [file 41598_2024_57410_MOESM1_ESM.pdf]

Table of Contents

Supplementary Table 1: SDOH Survey Development Process 3

Supplementary Table 2: SDOH Survey Modifications 5

Supplementary Methods: SDOH Scales and Scoring 6

Supplementary Table 4: Item non-response and Cronbach's Alpha for All of Us SDOH survey measures by participant characteristics 10

Supplementary Table 4A: Item non-response and Cronbach's alphas by participant characteristic for loneliness, social support, instrumental social support, and emotional support scales (N=117,783) 10

Supplementary Table 4B: Item non-response and Cronbach's alphas by participant characteristic for perceived stress, everyday discrimination, discrimination in healthcare settings, and social cohesion scales (N=117,783) 12

Supplementary Table 4C: Item non-response and Cronbach's alphas by participant characteristic for neighborhood physical disorder, neighborhood physical disorder, daily spiritual experiences, and PANES - Walking and bicycling scales[a] (N=117,783) 14

Supplementary Table 4D: Item non-response by participant characteristic for PANES - Crime and safety, food insecurity, housing instability, housing quality, and religious service attendance measures (N=117,783) 16

Supplementary Figure 5: Forest plots of multivariable logistic regression models predicting item non-response within SDOH scales. 18

Supplementary Figure A1. Odds of item non-response or incalculable score for the Loneliness Scale. 18

Supplementary Figure A2: Odds of item non-response or incalculable score for the Neighborhood Physical Disorder Scale. 19

Supplementary Figure A3: Odds of item non-response or incalculable score for the Neighborhood Social Disorder Scale. 20

Supplementary Figure A4: Odds of item non-response or incalculable score for the Everyday Discrimination Scale. 21

Supplementary Figure A5: Odds of item non-response or incalculable score for the Discrimination in Medical Settings Scale. 22

Supplementary Figure A6: Odds of item non-response or incalculable score for the Social Cohesion Scale. 23

Supplementary Figure A7: Odds of item non-response or incalculable score for the Perceived Stress Scale. 24

Supplementary Figure A8: Odds of item non-response or incalculable score for the Total Social Support Scale. 25

Supplementary Figure A9: Odds of item non-response or incalculable score for the Instrumental Social Support Scale 26

Supplementary Figure A10: Odds of item non-response or incalculable score for the Emotional Social Support Scale. 27

Supplementary Figure A11: Odds of item non-response or incalculable score for the Daily Spiritual Experiences Scale. 28

Supplementary Figure A12: Odds of item non-response or incalculable score for the Housing Instability Scale. 29

Supplementary Figure A13: Odds of item non-response or incalculable score for the Food Insecurity Scale. 30

Supplementary Figure A14: Odds of item non-response or incalculable score for the PANES - Walking and Bicycling Scale. 31

Supplementary Figure A15: Odds of item non-response or incalculable score for the PANES - Crime and Safety Scale. 32

Supplementary Figure A16: Odds of item non-response or incalculable score for the Housing Quality Scale. 33

Supplementary Figure A17: Odds of item non-response or incalculable score for the Religious Attendance Scale. 34

Supplementary Note: Acknowledgement List of Principal Investigators 35

Supplementary Table 1: SDOH Survey Development Process

Process	Considerations
Phase I: Select conceptual frameworks and define SDOH for the *All of Us* survey	• Prioritize frameworks that guide research on connections between social factors and health<br>• Prioritize frameworks and definitions that improve communication on social concepts in large and diverse participant audiences
Phase II: Define inclusion and exclusion criteria, and priorities for selecting and using constructs and measures	Inclusion criteria.<br>Concepts should:<br>• measure *perceptions* that can only be collected through participant responses;<br>• connect to core drivers of health inequities (e.g., perceived discrimination);<br>• have documentation on measure validation and psychometric performance;<br>• have strong use cases (structural, social and biologic) to facilitate research on mechanisms between SDOHs and health.<br>Exclusion criteria.<br>Concepts should not be:<br>• new concepts and measures without psychometric validation (with rare exception);<br>• concepts that can be collected without burdening participants (e.g., via geocoding)<br>• concepts that may be more reliability captured through other modalities besides participant surveys (e.g., wealth)<br>• concepts that may require sufficient items or measures to merit a dedicated survey (e.g., acculturation, wealth)<br>Priorities for selecting and incorporating measures.<br>• constructs and measures should be validated with high reliability in diverse cohorts and in multiple languages.<br>• measures should be included in the form in which they were validated, and item response sets should not be altered as practically possible.

Phase III: Review the science to select standardized measures with use cases in precision medicine	● Prioritize measures that operationalize concepts in the World Health Organization Conceptual Framework for Action on the Social Determinants of Health,[1] and the five domain areas of the Healthy People framework for SDOH (*social and community*; *economic stability*; *education*; *neighborhood and built environment*; *health and health care*.)[12]<br>● Prioritize measures with data from epidemiologic cohort studies and other designs that elucidate mechanisms among SDOHs and connections to health
Phase IV: Examine surveys, measures and items in other large biobanks, cohort studies, epidemiologic surveys and toolkits to find opportunities to align measures	● UK Biobank<br>● Million Veteran Program<br>● Behavioral Risk Factor Surveillance Survey<br>● National Health and Nutrition Examination Survey<br>● NIH PhenX Toolkit SDOH Collections
Phase V: Coordinate internally with other *All of Us* task forces to avoid duplication	● Mental health<br>● Environmental health<br>● "The Basics" assessment of income, educational attainment, race, ethnicity, age, sexual orientation, gender identity, health care access, insurance status
Phase VI: Consult with scientific subject matter experts and participant partners in *All of Us*	● Developers of key measures of interest<br>● NIH Institutes: NHLBI, NIDDK, NICHD, NIMHD, OBSSR, ORWH<br>● *All of Us* Participant Ambassadors

Supplementary Table 2: SDOH Survey Modifications

Survey modifications based on cognitive interview and *All of Us* Participant Ambassador feedback
Prioritized measures for inclusion and exclusion from the survey
Replaced mentions of “God” with “God (or a higher power)” in measures of religiousness and spirituality.
Added “I am not religious” as a response option in questions pertaining to God.
Added language in introductory text to better define the phrase “social determinants of health”
Re-ordered domains so that questions with negative framing do not all appear at the beginning of the survey.
Added “tool tip” at the beginning of each domain to clarify the scientific value of the survey questions.
Developed participant-facing frequently asked questions (FAQs) to help participants better understand SDOH in general, the topics included in the survey, and the role of SDOH in health and well-being. Some FAQs also include links to additional information for participants who are interested in learning more about SDOH and resources to assist any participants who may be in need of additional information or support in specific SDOH-related areas.

Supplementary Methods: SDOH Scales and Scoring

SDOH survey scoring documentation and recommendations are provided for the following scales:

Social Cohesion. The social cohesion scale connects to theories on neighborhood social organization.[34,35] The selected scale, also featured in the NIH PhenX toolkit, was modified and validated by Mujahid et al.[16] There are four items, rated on a 5-point agreement scale (1 = strongly agree, 2 = agree, 3 = neutral (neither agree nor disagree), 4 = disagree, and 5 = strongly disagree). The scoring is the mean of the four items. It was flagged as missing if any of the four items were missing a valid response. The original article reported a Cronbach's Alpha of 0.74, and a test-retest reliability of 0.65. Validation was done using census tract data.[16]

Social Support. The 8-item modified (mMOS-SS) social support scale from the Medical Outcomes Study was used.[17] The scale measures perceived social support. Each item is rated on a 5-point scale (1 = None of the time; 2 = A little of the time; 3 = Some of the time; 4 = Most of the time; 5 = All of the time). Three scores can be computed using an average of the items within the scale. A total social support scale can be computed using all eight items. It can be computed if 6 or more items have valid responses. The total score has a Cronbach's Alpha of 0.93. All 8-items had high item-total correlations. There are also two 4-item subscales, one to measure *instrumental support* (e.g., help prepare your meals), and the other to measure *emotional support* (e.g., someone to love and make you feel wanted). Separate reliability coefficients were not reported for the subscales. These scales are computed as averages and can be used if only a single item is missing, otherwise the scale is set to a missing value. Convergent and discriminant validity was demonstrated using correlations with self-report, demographic, and health measures.[17]

Loneliness. The 8-item UCLA Loneliness Scale (ULS-8) was used.[18] The items measured perceived lack of companionship and social isolation. The Loneliness scale uses a 4-point frequency rating to collect responses (1 = Never; 2 = Rarely; 3 = Sometimes; 4 = Often). The scale is scored by calculating an item mean and can be computed if 6 or more items have valid responses. Cronbach's Alpha of 0.84 was reported for the 8-item scale. Convergent validity was evaluated using correlations with other measures of mental health and interpersonal relationships.[18]

Everyday Discrimination. A 9-item everyday discrimination scale was developed by Williams et al.[19] It used a 6-point frequency scale (5 = Almost every day; 4 = At least once a week; 3 = A few times a month; 2 = A few times a year; 1 = Less than once a year; 0 = Never). Scoring is done by computing the mean item score. The scale is flagged as missing if more than two items do not have a valid score. The coefficient Alpha was 0.88 in the original study. The scale has been validated using self-report health, race, and measures of socioeconomic status.[36] There is an additional "check all that applies" to a descriptive item that asks participants "What do you think is the main reason for these experiences?" Response options are: 1. Your Ancestry or National Origins, 2. Your Gender, 3. Your Race, 4. Your Age, 5. Your Religion, 6. Your Height, 7. Your Weight, 8. Some other aspect of Your Physical Appearance, 9. Your Sexual orientation, 10. Your education or income level, 11. Other (specify). This item is not scored and is used as descriptive data.

Cohen Perceived Stress. The Cohen Perceived Stress Scale is a 10-item scale[22] (the original scale had 14 items)[21] that measures a participant's experience of everyday thoughts and feelings. The questions ask for a frequency rating in the past month using a 5-point scale (1 = Never; 2 = Almost Never; 3 = Sometimes; 4 = Fairly Often; 5 = Very Often). The scale is scored by multiplying the item mean times ten. It can be scored if more than 8 items have a valid response. Cronbach's Alpha for the original scale was reported to be 0.84 to 0.86.[21] Validation included associations with life events, depressive symptoms, and healthcare utilization.[21] The validated 10-item scale had a Cronbach's Alpha of 0.78.[22]

Daily Spiritual Experiences. The Daily Spiritual Experiences scale was adapted from the Brief Multidimensional Measure of Religiousness/Spirituality.[23] The Daily Spiritual Experiences is a single subscale from a longer instrument and includes items like "I feel God's presence." The items are rated using a 6-point scale (6 = Many times a day; 5 = Every day; 4 = Most days; 3 = Some days; 2 = Once in a while; 1 = Never or almost never). In response to *All of Us* participant feedback, this response set was altered to add additional choices: 0 = I do not believe in God (or a higher power) or 0 = I am not religious. The scale is scored as the average of the items and is flagged as missing if more than two items do not have a valid response. Cronbach's Alpha for the original scale is 0.91.[37] Validity data were not presented in the original paper, though the Fetzer Institute[37] report provides an extensive literature review.

Religious Service Attendance. One item on religious service attendance was obtained from the Women's Health study.[24] The wording of the item is "How often do you go to religious meetings or services?" The response options are: 5 = More than once a week; 4 = Once a week; 3 = 1 to 3 times per month; 2 = Less than once per month; 1 = Never (or almost never). In response to *All of Us* Participant feedback, a response option was added 0=I am not religious. There is no scoring for this item. Li et. al.[24] showed that religious attendance was a protective factor for all cause mortality. Due to a transcription error, the initial response set for this item was incorrectly displayed for 11,795 participants in the version 7 data. These observations are flagged as 'invalid' in version 7 data and combined with the 'PMI/Skip' category. Users should use the 'invalid' flag to identify these observations and can apply missing data techniques in the analysis of these cases.

Food Insecurity. A screening Food Insecurity measure based on the work of Hager et al[26] was included. There are two items (rated as often true, sometimes true or never true). The individual is considered food insecure if any item is rated as sometimes true or often true. High specificity and sensitivity has been reported in classifying families and food insecure using the two-item scale.[38] Convergent validity was demonstrated using logistic regression models of health outcomes.[26]

Housing Instability. A single item assessment of housing instability was included.[27] The item is "In the last 12 months, how many times have you or your family moved from one home to another?" Ash et al[39] used data on multiple address changes as a measure of housing insecurity/instability to inform payment models for assessing social risks.

Housing Quality. An item describing housing instability due to housing quality problems was used in the Accountable Health Communities Screening Tool.[28] The item is "Think about the place you live. Do you have problems with any of the following?" There are 8 check all that

apply response options (1 = Bug infestation; 2 = Mold; 3 = Lead paint or pipes; 4 = Inadequate heat; 5 = Oven or stove not working; 6 = No or not working smoke detectors; 7 = Water leaks; 8 = None of the above). In this report, it is scored as a binary variable and set to zero if option 8 is the only item selected, and to 1 if one or more of options 1-7 are selected. Data on reliability or validity were not available at the time of measure selection.

Neighborhood Physical and Social Disorder. The Neighborhood Physical and Social Disorder scale is from the work of Ross and Mirowsky.[29] The Physical Disorder items reflect negative physical characteristics such as noise, graffiti, and abandoned buildings. The Neighborhood Physical Disorder Scale has six items, with two items reverse scored. Responses are collected using a 4-point agreement scale (1 = strongly disagree; 2 = disagree; 3 = agree; 4 = strongly agree). Scoring is the average of the items, with the score set to missing if more than one item is not a valid response. Neighborhood Social Disorder measures psychosocial problems associated with the people in one's neighborhood and their behavior (perceived safety, trouble with neighbors). There are seven items in the scale, and it uses a 4-point agreement rating scale (1 = strongly disagree; 2 = disagree; 3 = agree; 4 = strongly agree). The scale is scored by averaging the ratings of the 7 items. The scale is set to missing if more than one item does not have a valid response. Coefficient alpha was 0.92 for the combined physical and social neighborhood disorder measure. The scale was validated against census tract data, health outcomes, and sociodemographic characteristics.[29]

Physical Activity and Neighborhood Environment Scale (PANES) – Walking and Bicycling. The Walking and Bicycling Scale of the PANES was adapted from Sallis et al[30] and consists of five items that are rated on a 4-point agreement scale (1 = Strongly disagree; 2 = Somewhat disagree; 3 = Somewhat agree; 4 = Strongly agree; Missing= Skip/Don't know. Sallis et al[30] reported adequate test-retest reliability. In this technical report, we scored this scale as the average of the five items and flagged the score as missing if more than one item does not have a valid response. Two items related to walking and bicycling include the response category "Does not apply to my neighborhood." As an alternative to deleting the "Does not apply to my neighborhood" responses, re-scoring this response category as "1 = Strongly disagree/Does not apply to my neighborhood" results in good internal consistency reliability in *All of Us* (Cronbach's alpha =0.78) and may allow the ability to retain observations in rural geographies.

Physical Activity and Neighborhood Environment Survey (PANES) – Crime Safety. Two items from the original 17-item PANES scale that represent perceptions of neighborhood safety from crime were selected.[30] The items are rated on a 4-point agreement scale (1= Strongly disagree; 2 = Somewhat disagree; 3 = Somewhat agree; 4 = Strongly agree; Missing= Skip/Don't know). Test-retest correlations were reported for individual items and showed acceptable reliability.[30] We reversed and averaged these items to form a PANES-Crime Safety measure. If either item is not a valid response (including Skip/Don't know), we flagged the scale as missing. Additional coding strategies for individual items are recommended by Sallis et al.[40]

Physical Activity and Neighborhood Environment Scale (PANES) – Perceived Residential Density/Neighborhood Housing Type. Sallis et al[30] developed a series of self-report measure perceptions of the characteristics of the neighborhood environment that can influence physical

activity. All of the PANES measures are introduced with the prompt, “Think about the different facilities in and around your neighborhood. By this we mean the area ALL around your home that you could walk to in 10-15 minutes.” Perceived residential density is measured with an indicator variable: “What is the main type of housing in your neighborhood?” There are 5 response choices: “Detached single-family housing; Townhouses, row houses, apartments, or condos of 2-3 stories; Mix of single-family residences and townhouses, row houses, apartments or condos; Apartments or condos of 4-12 stories; and Apartments or condos of more than 12 stories. Interpretation of this item and coding strategies are provided by Sallis et al.[30,40]

Discrimination in Health Care Settings. A 7-item scale to measure experiences of discrimination in health care settings was obtained from Peek et al.[32] It asks for frequency ratings on a 5-point scale (1 = Never; 2 = Rarely; 3 = Sometimes; 4 = Most of the time; 5 = Always) of how one is treated in health care settings (e.g. You receive poorer service than others). Scoring the scales is done by computing the item average, and the scale is flagged as missing if more than two items do not have valid responses. Cronbach’s Alpha was 0.89, and the test-retest reliability was 0.58. The scale was validated using other measures of discrimination and had low correlations with depression and social desirability.[32]

Supplementary Table 4: Item non-response and Cronbach's Alpha for All of Us SDOH survey measures by participant characteristics

Supplementary Table 4A: Item non-response and Cronbach's alphas by participant characteristic for loneliness, social support, instrumental social support, and emotional support scales (N=117,783)

	Loneliness		Social support		Instrumental social support		Emotional social support	
	Item non-response	Alpha	Item non-response	Alpha	Item non-response	Alpha	Item non-response	Alpha
Total	3018 (2.6%)	0.87	3421 (2.9%)	0.95	2088 (1.8%)	0.95	2680 (2.3%)	0.91
RBR Overall	433 (1.7%)	0.87	285 (1.1%)	0.94	150 (0.6%)	0.96	227 (0.9%)	0.90
UBR Overall	2768 (3.0%)	0.87	3136 (3.4%)	0.95	1938 (2.1%)	0.95	2453 (2.7%)	0.91
Racial identity[a]								
White	1790 (2.0%)	0.87	2105 (2.4%)	0.95	1174 (1.3%)	0.95	1597 (1.8%)	0.91
Black, African or African American	455 (5.1%)	0.83	524 (5.8%)	0.95	356 (4.0%)	0.95	435 (4.8%)	0.91
Hispanic/Latino/Spanish	457 (5.7%)	0.85	435 (5.5%)	0.95	319 (4.0%)	0.95	362 (4.5%)	0.91
Asian	58 (1.9%)	0.87	69 (2.2%)	0.95	45 (1.4%)	0.95	58 (1.9%)	0.92
Native Hawaiian or Pacific Islander (NHPI)[b]								
Middle Eastern or North African (MENA)[b]								
Multi-Racial	67 (1.6%)	0.87	67 (1.6%)	0.95	40 (1.0%)	0.96	58 (1.4%)	0.91
None of these describe me	29 (2.8%)	0.87	39 (3.8%)	0.94	26 (2.5%)	0.95	37 (3.6%)	0.91
Prefer not to answer or skip	148 (3.7%)	0.87	159 (3.9%)	0.95	108 (2.7%)	0.95	120 (3.0%)	0.91
Sex assigned at birth								
Female	1839 (2.5%)	0.87	2064 (2.8%)	0.95	1189 (1.6%)	0.95	1651 (2.2%)	0.91
Male	1094 (2.7%)	0.87	1256 (3.1%)	0.95	833 (2.1%)	0.96	937 (2.3%)	0.91
Intersex, none of these describe me, prefer not to answer, or skip	85 (2.9%)	0.87	100 (3.4%)	0.95	66 (2.2%)	0.95	91 (3.1%)	0.91
Gender identity								
Woman	1831 (2.5%)	0.87	2069 (2.8%)	0.95	1195 (1.6%)	0.95	1655 (2.3%)	0.91
Man	1092 (2.7%)	0.87	1258 (3.1%)	0.95	831 (2.1%)	0.96	948 (2.4%)	0.91
Non-Binary[b]								
Transgender[b]								
None of these describe me, prefer not to answer, or skip	91 (2.5%)	0.88	87 (2.4%)	0.95	58 (1.6%)	0.95	73 (2.0%)	0.91
Sexual orientation								
Straight	2654 (2.6%)	0.87	3032 (3.0%)	0.95	1832 (1.8%)	0.95	2370 (2.3%)	0.91
Lesbian[b]								
Gay	55 (1.8%)	0.88	61 (2.0%)	0.95	40 (1.3%)	0.96	47 (1.6%)	0.91
Bisexual	50 (1.1%)	0.86	69 (1.5%)	0.94	42 (0.9%)	0.95	51 (1.1%)	0.91

Supplementary Table 4A: Item non-response and Cronbach's alphas by participant characteristic for loneliness, social support, instrumental social support, and emotional support scales (N=117,783)								
	Loneliness		Social support		Instrumental social support		Emotional social support	
	Item non-response	Alpha	Item non-response	Alpha	Item non-response	Alpha	Item non-response	Alpha
Multiple[b]								
None of these describe me	76 (3.2%)	0.86	75 (3.2%)	0.94	55 (2.4%)	0.95	61 (2.7%)	0.90
Prefer not to answer or skip	150 (4.0%)	0.87	152 (4.1%)	0.95	102 (2.7%)	0.95	124 (3.3%)	0.91
Educational attainment								
College graduate or advanced degree	1374 (1.9%)	0.87	1594 (2.2%)	0.95	909 (1.2%)	0.95	1212 (1.7%)	0.91
College (Years One to Three)	752 (2.7%)	0.88	887 (3.2%)	0.95	530 (1.9%)	0.95	692 (2.5%)	0.91
Grade Twelve or GED	517 (4.8%)	0.86	564 (5.3%)	0.95	376 (3.5%)	0.95	470 (4.4%)	0.91
Less than high school degree or equivalent	221 (8.6%)	0.83	218 (8.4%)	0.94	154 (6.0%)	0.93	185 (7.2%)	0.91
Prefer not to answer or skip	154 (4.5%)	0.86	158 (4.6%)	0.95	119 (3.5%)	0.95	121 (3.5%)	0.91
Income								
>150k	261 (1.2%)	0.86	352 (1.7%)	0.94	173 (0.8%)	0.95	265 (1.3%)	0.90
100k-150k	294 (1.6%)	0.86	356 (1.9%)	0.94	194 (1.0%)	0.95	257 (1.4%)	0.90
50k-100k	682 (2.2%)	0.86	774 (2.5%)	0.94	453 (1.5%)	0.95	568 (1.8%)	0.90
<50k	1086 (3.3%)	0.87	1227 (3.8%)	0.95	762 (2.3%)	0.95	1001 (3.1%)	0.91
Prefer not to answer or skip	695 (4.9%)	0.86	712 (5.0%)	0.95	506 (3.6%)	0.95	589 (4.1%)	0.91
Disability								
No disabilities identified	1144 (2.2%)	0.86	1292 (2.5%)	0.95	793 (1.5%)	0.96	995 (1.9%)	0.91
Living with disabilities	500 (3.2%)	0.87	569 (3.6%)	0.94	355 (2.3%)	0.95	442 (2.8%)	0.91
Not assessed[c]	1374 (2.7%)	0.87	1560 (3.1%)	0.95	939 (1.9%)	0.95	1243 (2.5%)	0.91
Survey language								
English	2743 (2.4%)	0.87	3175 (2.8%)	0.95	1908 (1.7%)	0.95	2468 (2.1%)	0.91
Spanish	275 (10.6%)	0.81	245 (9.5%)	0.94	179 (7.0%)	0.93	212 (8.2%)	0.90

Abbreviations: RBR, represented in biomedical research; UBR, underrepresented in biomedical research

[a]Racial and ethnic identity: racial identity (Asian, H/L/S, White, etc.); ethnic identity (Japanese, Cuban, Irish, etc.). Ethnic identity data not provided due to space limitations.

[b]Cells with counts below 20 were suppressed; counts were also suppressed to prevent participant re-identification due to small sample sizes (NHPI, MENA, Non-Binary, Transgender, Lesbian, and Multiple Sexual Orientation)

[c]Participants that responded to the Basics survey before October 22, 2019 do not have demographic information on disability

Supplementary Table 4B: Item non-response and Cronbach's alphas by participant characteristic for perceived stress, everyday discrimination, discrimination in healthcare settings, and social cohesion scales (N=117,783)

	Perceived stress		Everyday discrimination		Discrimination in healthcare settings		Social cohesion	
	Item non-response	Alpha	Item non-response	Alpha	Item non-response	Alpha	Item non-response	Alpha
Total	7129 (6.1%)	0.91	3915 (3.3%)	0.91	2885 (2.5%)	0.86	4770 (4.1%)	0.87
RBR Overall	693 (2.7%)	0.90	314 (1.2%)	0.89	267 (1.1%)	0.89	145 (0.6%)	0.86
UBR Overall	6436 (7.0%)	0.91	3601 (3.9%)	0.91	2618 (2.8%)	0.90	1731 (1.9%)	0.87
Racial identity[a]								
White	4887 (5.6%)	0.91	2427 (2.8%)	0.90	1685 (1.9%)	0.89	3270 (3.7%)	0.87
Black, African or African American	843 (9.4%)	0.87	577 (6.4%)	0.92	441 (4.9%)	0.91	551 (6.1%)	0.86
Hispanic/Latino/Spanish	660 (8.3%)	0.87	499 (6.3%)	0.91	433 (5.4%)	0.90	484 (6.1%)	0.87
Asian	143 (4.6%)	0.89	73 (2.3%)	0.91	53 (1.7%)	0.90	75 (2.4%)	0.86
Native Hawaiian or Pacific Islander (NHPI) [b]								
Middle Eastern or North African (MENA)[b]								
Multi-Racial	156 (3.7%)	0.91	89 (2.1%)	0.92	73 (1.7%)	0.90	99 (2.4%)	0.86
None of these describe me	72 (6.9%)	0.91	53 (5.1%)	0.92	38 (3.7%)	0.90	55 (5.3%)	0.88
Prefer not to answer or skip	336 (8.3%)	0.91	184 (4.6%)	0.91	139 (3.5%)	0.90	215 (5.3%)	0.86
Sex assigned at birth								
Female	4359 (5.9%)	0.91	2463 (3.3%)	0.91	1814 (2.4%)	0.89	2860 (3.8%)	0.87
Male	2571 (6.4%)	0.89	1324 (3.3%)	0.92	970 (2.4%)	0.90	1758 (4.4%)	0.86
Intersex, none of these describe me, prefer not to answer, or skip	199 (6.7%)	0.91	128 (4.3%)	0.92	100 (3.4%)	0.89	151 (5.1%)	0.87
Gender identity								
Woman	4337 (5.9%)	0.91	2464 (3.4%)	0.91	1803 (2.5%)	0.89	2842 (3.9%)	0.87
Man	2563 (6.4%)	0.89	1327 (3.3%)	0.91	977 (2.4%)	0.90	1757 (4.4%)	0.86
Non-Binary[b]								
Transgender[b]								
None of these describe me, prefer not to answer, or skip	214 (6.0%)	0.92	113 (3.2%)	0.93	97 (2.7%)	0.91	153 (4.3%)	0.86
Sexual orientation								
Straight	6385 (6.3%)	0.90	3461 (3.4%)	0.91	2536 (2.5%)	0.89	4244 (4.2%)	0.87
Lesbian[b]								
Gay	137 (4.5%)	0.91	62 (2.0%)	0.92	41 (1.3%)	0.90	107 (3.5%)	0.85
Bisexual	140 (3.0%)	0.92	78 (1.7%)	0.90	66 (1.4%)	0.90	89 (1.9%)	0.85
Multiple[b]								

Supplementary Table 4B: Item non-response and Cronbach's alphas by participant characteristic for perceived stress, everyday discrimination, discrimination in healthcare settings, and social cohesion scales (N=117,783)

	Perceived stress		Everyday discrimination		Discrimination in healthcare settings		Social cohesion	
	Item non-response	Alpha	Item non-response	Alpha	Item non-response	Alpha	Item non-response	Alpha
None of these describe me	98 (4.2%)	0.91	88 (3.8%)	0.92	66 (2.8%)	0.91	77 (3.3%)	0.83
Prefer not to answer or skip	288 (7.7%)	0.90	186 (5.0%)	0.92	149 (4.0%)	0.90	195 (5.2%)	0.86
Educational attainment								
College graduate or advanced degree	3865 (5.3%)	0.91	1790 (2.4%)	0.90	1261 (1.7%)	0.89	2519 (3.4%)	0.86
College (Years One to Three)	1700 (6.2%)	0.91	1021 (3.7%)	0.91	754 (2.8%)	0.90	1189 (4.3%)	0.88
Grade Twelve or GED	1000 (9.4%)	0.89	683 (6.4%)	0.92	508 (4.8%)	0.90	651 (6.1%)	0.88
Less than high school degree or equivalent	301 (11.6%)	0.84	249 (9.7%)	0.92	212 (8.2%)	0.90	234 (9.1%)	0.87
Prefer not to answer or skip	263 (7.6%)	0.90	171 (5.0%)	0.91	149 (4.3%)	0.89	177 (5.1%)	0.87
Income								
>150k	894 (4.2%)	0.89	336 (1.6%)	0.89	256 (1.2%)	0.89	575 (2.7%)	0.85
100k-150k	835 (4.5%)	0.90	368 (2.0%)	0.89	260 (1.4%)	0.89	601 (3.2%)	0.85
50k-100k	1768 (5.7%)	0.91	872 (2.8%)	0.89	614 (2.0%)	0.90	1150 (3.7%)	0.86
<50k	2320 (7.1%)	0.90	1456 (4.5%)	0.90	1058 (3.2%)	0.89	1580 (4.8%)	0.87
Prefer not to answer or skip	1312 (9.2%)	0.90	883 (6.2%)	0.90	697 (4.9%)	0.90	864 (6.1%)	0.87
Disability								
No disabilities identified	2821 (5.4%)	0.90	1424 (2.7%)	0.90	1087 (2.1%)	0.89	1820 (3.5%)	0.87
Living with disabilities	1102 (7.0%)	0.91	670 (4.3%)	0.91	483 (3.1%)	0.90	793 (5.1%)	0.87
Not assessed[c]	3206 (6.4%)	0.91	1821 (3.6%)	0.91	1315 (2.6%)	0.90	2157 (4.3%)	0.86
Survey language								
English	6805 (5.9%)	0.91	3617 (3.1%)	0.87	2651 (2.3%)	0.90	4495 (3.9%)	0.87
Spanish	324 (12.5%)	0.86	298 (11.5%)	0.83	234 (9.1%)	0.88	275 (10.6%)	0.86

Abbreviations: RBR, represented in biomedical research; UBR, underrepresented in biomedical research
[a]Racial and ethnic identity: racial identity (Asian, H/L/S, White, etc.); ethnic identity (Japanese, Cuban, Irish, etc.). Ethnic identity data not provided due to space limitations
[b]Cells with counts below 20 were suppressed; counts were also suppressed to prevent participant re-identification due to small sample sizes (NHPI, MENA, Non-Binary, Transgender, Lesbian, and Multiple Sexual Orientation)
[c]Participants that responded to the Basics survey before October 22, 2019 do not have demographic information on disability

Supplementary Table 4C: Item non-response and Cronbach's alphas by participant characteristic for neighborhood physical disorder, neighborhood physical disorder, daily spiritual experiences, and PANES - Walking and bicycling scales[a] (N=117,783)

	Neighborhood physical disorder		Neighborhood social disorder		Daily spiritual experiences		PANES - Walking and Bicycling	
	Item non-response	Alpha	Item non-response	Alpha	Item non-response	Alpha	Item non-response	Alpha
Total	3417 (2.9%)	0.84	6215 (5.3%)	0.87	1334 (1.1%)	0.81	5771 (4.9%)	0.78
RBR Overall	263 (1.0%)	0.83	567 (2.2%)	0.85	143 (0.6%)	0.81	657 (2.6%)	0.80
UBR Overall	3153 (3.4%)	0.84	5648 (6.1%)	0.87	1191 (1.3%)	0.82	5114 (5.5%)	0.78
Racial identity [b]								
White	2157 (2.5%)	0.82	4139 (4.7%)	0.86	684 (0.8%)	0.81	3898 (4.4%)	0.79
Black, African or African American	446 (5.0%)	0.84	763 (8.5%)	0.88	248 (2.8%)	0.87	702 (7.8%)	0.74
Hispanic/Latino/Spanish	492 (6.2%)	0.84	676 (8.5%)	0.87	217 (2.7%)	0.84	552 (6.9%)	0.73
Asian	58 (1.9%)	0.85	105 (3.4%)	0.86	39 (1.3%)	0.79	118 (3.8%)	0.70
Native Hawaiian or Pacific Islander (NHPI) [c]								
Middle Eastern or North African (MENA)								
Multi-Racial	66 (1.6%)	0.85	124 (2.9%)	0.87	47 (1.1%)	0.79	159 (3.8%)	0.74
None of these describe me	30 (2.9%)	0.84	72 (6.9%)	0.88	22 (2.1%)	0.81	55 (5.3%)	0.78
Prefer not to answer or skip	152 (3.8%)	0.84	304 (7.5%)	0.87	63 (1.6%)	0.81	256 (6.4%)	0.78
Sex assigned at birth								
Female	2156 (2.9%)	0.84	3936 (5.3%)	0.87	784 (1.1%)	0.81	3682 (5.0%)	0.79
Male	1158 (2.9%)	0.83	2088 (5.2%)	0.87	506 (1.3%)	0.82	1903 (4.7%)	0.78
Intersex, none of these describe me, prefer not to answer, or skip	103 (3.5%)	0.85	191 (6.4%)	0.87	44 (1.5%)	0.81	186 (6.3%)	0.78
Gender identity								
Woman	2143 (2.9%)	0.84	3898 (5.3%)	0.87	775 (1.1%)	0.81	3652 (5.0%)	0.79
Man	1160 (2.9%)	0.83	2084 (5.2%)	0.87	509 (1.3%)	0.82	1893 (4.7%)	0.78
Non-Binary								
Transgender								
None of these describe me, prefer not to answer, or skip	109 (3.1%)	0.85	218 (6.1%)	0.87	46 (1.3%)	0.79	195 (5.5%)	0.77
Sexual orientation								
Straight	3017 (3.0%)	0.83	5506 (5.4%)	0.87	1159 (1.1%)	0.82	4940 (4.9%)	0.79
Lesbian								
Gay	66 (2.2%)	0.84	125 (4.1%)	0.87	20 (0.7%)	0.79	146 (4.8%)	0.77
Bisexual	65 (1.4%)	0.86	112 (2.4%)	0.87	32 (0.7%)	0.77	181 (3.9%)	0.78

Supplementary Table 4C: Item non-response and Cronbach's alphas by participant characteristic for neighborhood physical disorder, neighborhood physical disorder, daily spiritual experiences, and PANES - Walking and bicycling scales[a] (N=117,783)

	Neighborhood physical disorder		Neighborhood social disorder		Daily spiritual experiences		PANES - Walking and Bicycling	
	Item non-response	Alpha	Item non-response	Alpha	Item non-response	Alpha	Item non-response	Alpha
Multiple								
None of these describe me	76 (3.2%)	0.83	116 (5.0%)	0.85	30 (1.3%)	0.77	138 (5.9%)	0.77
Prefer not to answer or skip	156 (4.2%)	0.84	281 (7.5%)	0.87	80 (2.1%)	0.81	275 (7.3%)	0.77
Educational attainment								
College graduate or advanced degree	1541 (2.1%)	0.83	3207 (4.4%)	0.85	535 (0.7%)	0.80	2917 (4.0%)	0.78
College (Years One to Three)	915 (3.3%)	0.84	1615 (5.9%)	0.88	330 (1.2%)	0.83	1490 (5.4%)	0.79
Grade Twelve or GED	563 (5.3%)	0.84	847 (7.9%)	0.88	275 (2.6%)	0.85	837 (7.8%)	0.80
Less than high school degree or equivalent	245 (9.5%)	0.81	299 (11.6%)	0.86	109 (4.2%)	0.86	280 (10.8%)	0.80
Prefer not to answer or skip	152 (4.4%)	0.85	246 (7.2%)	0.87	85 (2.5%)	0.82	246 (7.2%)	0.78
Income								
>150k	303 (1.4%)	0.82	695 (3.3%)	0.83	94 (0.5%)	0.80	585 (2.8%)	0.79
100k-150k	328 (1.8%)	0.81	714 (3.8%)	0.84	89 (0.5%)	0.81	667 (3.6%)	0.78
50k-100k	768 (2.5%)	0.82	1464 (4.7%)	0.85	229 (0.7%)	0.82	1315 (4.3%)	0.79
<50k	1267 (3.9%)	0.84	2131 (6.5%)	0.88	474 (1.5%)	0.82	2039 (6.2%)	0.78
Prefer not to answer or skip	751 (5.3%)	0.83	1211 (8.5%)	0.87	447 (3.1%)	0.83	1165 (8.2%)	0.78
Disability								
No disabilities identified	1246 (2.4%)	0.84	2283 (4.4%)	0.87	577 (1.1%)	0.81	2210 (4.3%)	0.79
Living with disabilities	596 (3.8%)	0.83	977 (6.2%)	0.88	197 (1.3%)	0.82	942 (6.0%)	0.78
NA[d]	1574 (3.1%)	0.83	2955 (5.9%)	0.87	560 (1.1%)	0.81	2619 (5.2%)	0.78
Survey language								
English	3108 (2.7%)	0.83	5813 (5.1%)	0.87	1241 (1.1%)	0.81	5495 (4.8%)	0.79
Spanish	309 (11.9%)	0.80	402 (15.5%)	0.83	93 (3.6%)	0.85	276 (10.7%)	0.74

Abbreviations: PANES, Physical Activity and Neighborhood Environment Scale; RBR, represented in biomedical research; UBR, underrepresented in biomedical research

[a]For PANES items related to walking and bicycling, respondents who answered this item “does not apply to my neighborhood” were not included in score distributions and were not counted in non-response totals.

[b]Racial and ethnic identity: racial identity (Asian, H/L/S, White, etc.); ethnic identity (Japanese, Cuban, Irish, etc.). Ethnic identity data not provided due to space limitations

[c]Cells with counts below 20 were suppressed; counts were also suppressed to prevent participant re-identification due to small sample sizes (NHPI, MENA, Non-Binary, Transgender, Lesbian, and Multiple Sexual Orientation)

[d]Participants that responded to the Basics survey before October 22, 2019 do not have demographic information on disability

Supplementary Table 4D: Item non-response by participant characteristic for PANES - Crime and safety, food insecurity, housing instability, housing quality, and religious service attendance measures (N=117,783)					
	**PANES - Crime and Safety**	**Food insecurity**	**Housing instability**	**Housing quality**	**Religious service attendance[a]**
Total	15,293 (13.0%)	1542 (1.3%)	3620 (3.1%)	6134 (5.2%)	1825 (1.5%)
RBR Overall	2040 (8.0%)	127 (0.5%)	327 (1.3%)	651 (2.6%)	180 (0.7%)
UBR Overall	13,253 (14.4%)	1415 (1.5%)	3293 (3.6%)	5483 (5.9%)	1645 (1.8%)
Racial identity[b]					
White	10,636 (12.1%)	775 (0.9%)	2052 (2.3%)	4664 (5.3%)	973 (1.1%)
Black, African or African American	1617 (18.0%)	295 (3.3%)	593 (6.6%)	419 (4.7%)	332 (3.7%)
Hispanic/Latino/Spanish	1363 (17.1%)	258 (3.2%)	556 (7.0%)	412 (5.2%)	283 (3.6%)
Asian	347 (11.1%)	43 (1.4%)	90 (2.9%)	126 (4.0%)	50 (1.6%)
Native Hawaiian or Pacific Islander (NHPI)[c]					
Middle Eastern or North African (MENA)[c]					
Multi-Racial	443 (10.5%)	52 (1.2%)	103 (2.4%)	141 (3.3%)	58 (1.4%)
None of these describe me[c]	171 (16.5%)		48 (4.6%)	50 (4.8%)	31 (3.0%)
Prefer not to answer or skip	637 (15.8%)	85 (2.1%)	149 (3.7%)	295 (7.3%)	84 (2.1%)
Sex assigned at birth					
Female	9902 (13.3%)	908 (1.2%)	2085 (2.8%)	3594 (4.8%)	1101 (1.5%)
Male	4998 (12.4%)	575 (1.4%)	1435 (3.6%)	2367 (5.9%)	658 (1.6%)
Intersex, none of these describe me, prefer not to answer, or skip	393 (13.2%)	59 (2.0%)	100 (3.4%)	173 (5.8%)	66 (2.2%)
Gender identity					
Woman	9814 (13.4%)	901 (1.2%)	2079 (2.8%)	3574 (4.9%)	1094 (1.5%)
Man	4981 (12.5%)	580 (1.5%)	1426 (3.6%)	2365 (5.9%)	662 (1.7%)
Non-Binary[c]					
Transgender[c]					
None of these describe me, prefer not to answer, or skip	433 (12.1%)	56 (1.6%)	109 (3.0%)	182 (5.1%)	63 (1.8%)
Sexual orientation					
Straight	13493 (13.2%)	1326 (1.3%)	3183 (3.1%)	5525 (5.4%)	1578 (1.6%)
Lesbian[c]					
Gay	321 (10.5%)	28 (0.9%)	60 (2.0%)	113 (3.7%)	32 (1.1%)
Bisexual	403 (8.7%)	38 (0.8%)	72 (1.6%)	136 (2.9%)	47 (1.0%)
Multiple[c]					

Supplementary Table 4D: Item non-response by participant characteristic for PANES - Crime and safety, food insecurity, housing instability, housing quality, and religious service attendance measures (N=117,783)					
	**PANES - Crime and Safety**	**Food insecurity**	**Housing instability**	**Housing quality**	**Religious service attendance**[a]
None of these describe me	302 (12.9%)	60 (1.9%)	97 (3.1%)	71 (3.0%)	44 (1.9%)
Prefer not to answer or skip	552 (14.7%)	86 (2.0%)	203 (4.7%)	224 (6.0%)	104 (2.8%)
Educational attainment					
College graduate or advanced degree	8671 (11.8%)	604 (0.8%)	1581 (2.2%)	3835 (5.2%)	743 (1.0%)
College (Years One to Three)	3774 (13.8%)	388 (1.4%)	932 (3.4%)	1331 (4.9%)	474 (1.7%)
Grade Twelve or GED	1832 (17.1%)	327 (3.1%)	643 (5.9%)	619 (5.8%)	364 (3.4%)
Less than high school degree or equivalent	551 (21.3%)	129 (5.0%)	292 (11.3%)	139 (5.4%)	137 (5.3%)
Prefer not to answer or skip	465 (13.5%)	94 (2.7%)	181 (5.2%)	210 (6.1%)	107 (3.1%)
Income					
>150k	2108 (9.9%)	106 (0.5%)	338 (1.6%)	995 (4.7%)	137 (0.7%)
100k-150k	1942 (10.4%)	110 (0.6%)	305 (1.6%)	844 (4.5%)	151 (0.8%)
50k-100k	3897 (12.6%)	273 (0.9%)	752 (2.4%)	1663 (5.4%)	342 (1.1%)
<50k	4895 (15.0%)	606 (1.9%)	1340 (4.1%)	1616 (4.9%)	687 (2.1%)
Prefer not to answer or skip	2451 (17.2%)	447 (3.1%)	885 (6.2%)	1016 (7.1%)	508 (3.6%)
Disability					
No disabilities identified	6277 (12.1%)	641 (1.2%)	1284 (2.5%)	2439 (4.7%)	728 (1.4%)
Living with disabilities	2253 (14.4%)	282 (1.8%)	589 (3.8%)	744 (4.8%)	326 (2.1%)
Not assessed[d]	6763 (13.5%)	619 (1.2%)	1747 (3.5%)	2951 (5.9%)	771 (1.5%)
Survey language					
English	14630 (12.7%)	1399 (1.2%)	3296 (2.9%)	5955 (5.2%)	1703 (1.5%)
Spanish	662 (25.6%)	143 (5.5%)	324 (12.5%)	179 (6.9%)	122 (4.7%)

Abbreviations: PANES, Physical Activity and Neighborhood Environment Scale; RBR, represented in biomedical research; UBR, underrepresented in biomedical research

[a]Participants who responded to the incorrect response set for the Religious Service Attendance item (N=11,795) are flagged as 'invalid' in version 7 data; these respondents are not included in item non-response calculations.

[b]Racial and ethnic identity: racial identity (Asian, H/L/S, White, etc.); ethnic identity (Japanese, Cuban, Irish, etc.). Ethnic identity data not provided due to space limitations

[c]Cells with counts below 20 were suppressed; counts were also suppressed to prevent participant re-identification due to small sample sizes (NHPI, MENA, None of these describe me race, Non-Binary, Transgender, Lesbian, and Multiple Sexual Orientation)

[d]Participants that responded to the Basics survey before October 22, 2019 do not have demographic information on disability

Supplementary Figure 5: Forest plots of multivariable logistic regression models predicting item non-response within SDOH scales.

Supplementary Figure A1. Odds of item non-response or incalculable score for the Loneliness Scale.

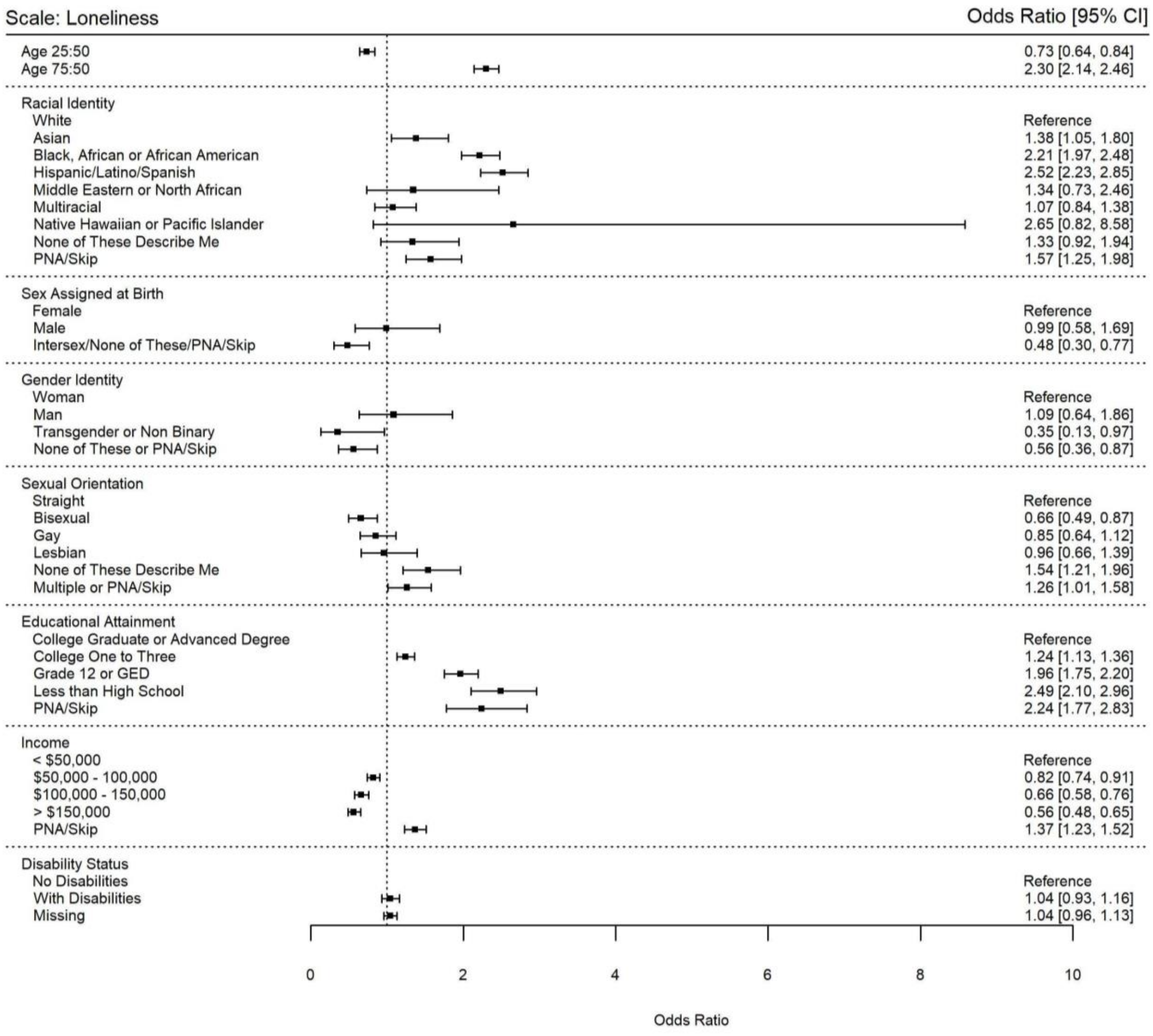

Abbreviations: MENA, Middle Eastern or North African; NHPI, Native Hawaiian or Pacific Islander; PNA, prefer not to answer
Sex at Birth: Sex Assigned at Birth

Supplementary Figure A2: Odds of item non-response or incalculable score for the Neighborhood Physical Disorder Scale.

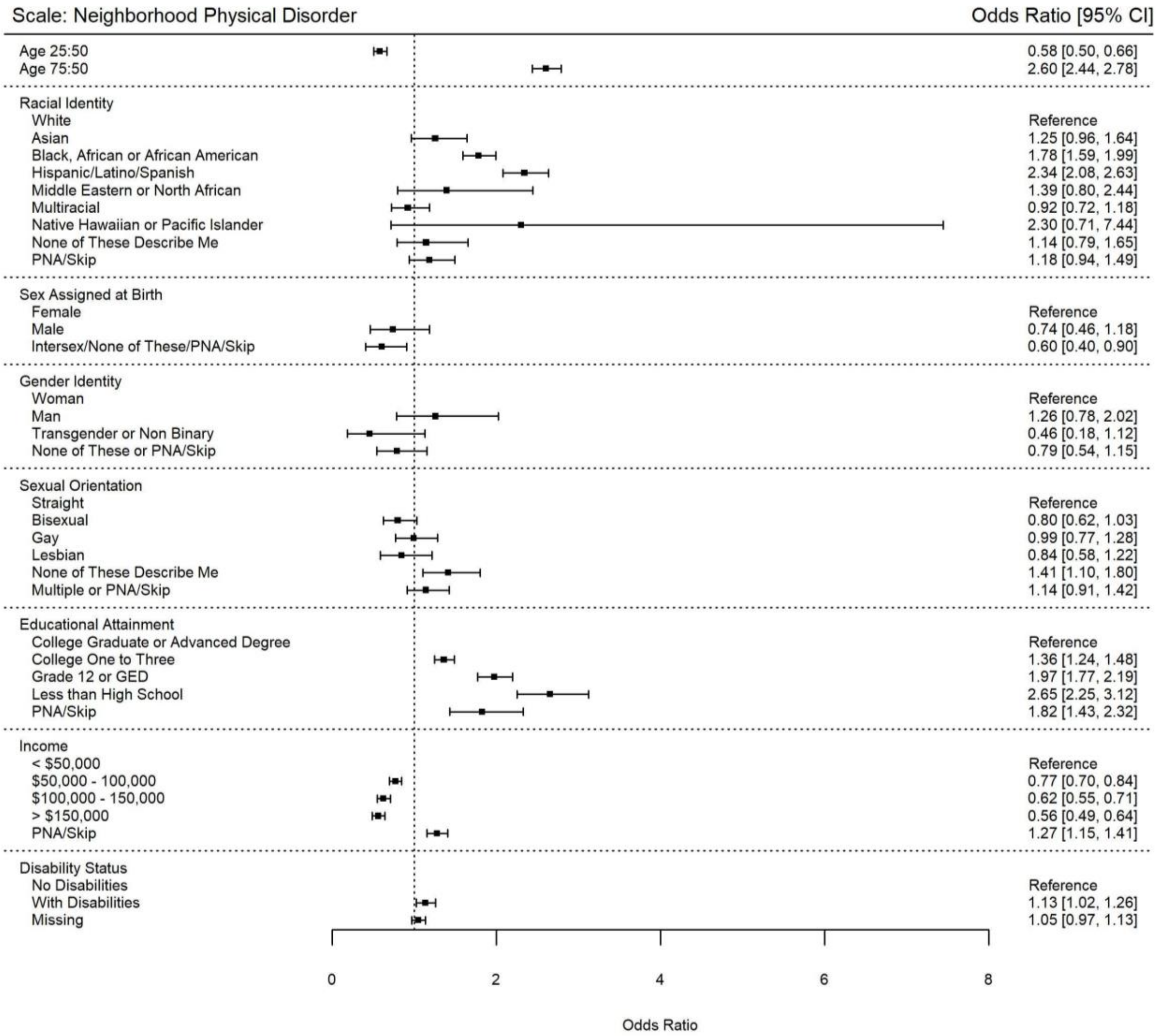

Abbreviations: MENA, Middle Eastern or North African; NHPI, Native Hawaiian or Pacific Islander; PNA, prefer not to answer
Sex at Birth: Sex Assigned at Birth

Supplementary Figure A3: Odds of item non-response or incalculable score for the Neighborhood Social Disorder Scale.

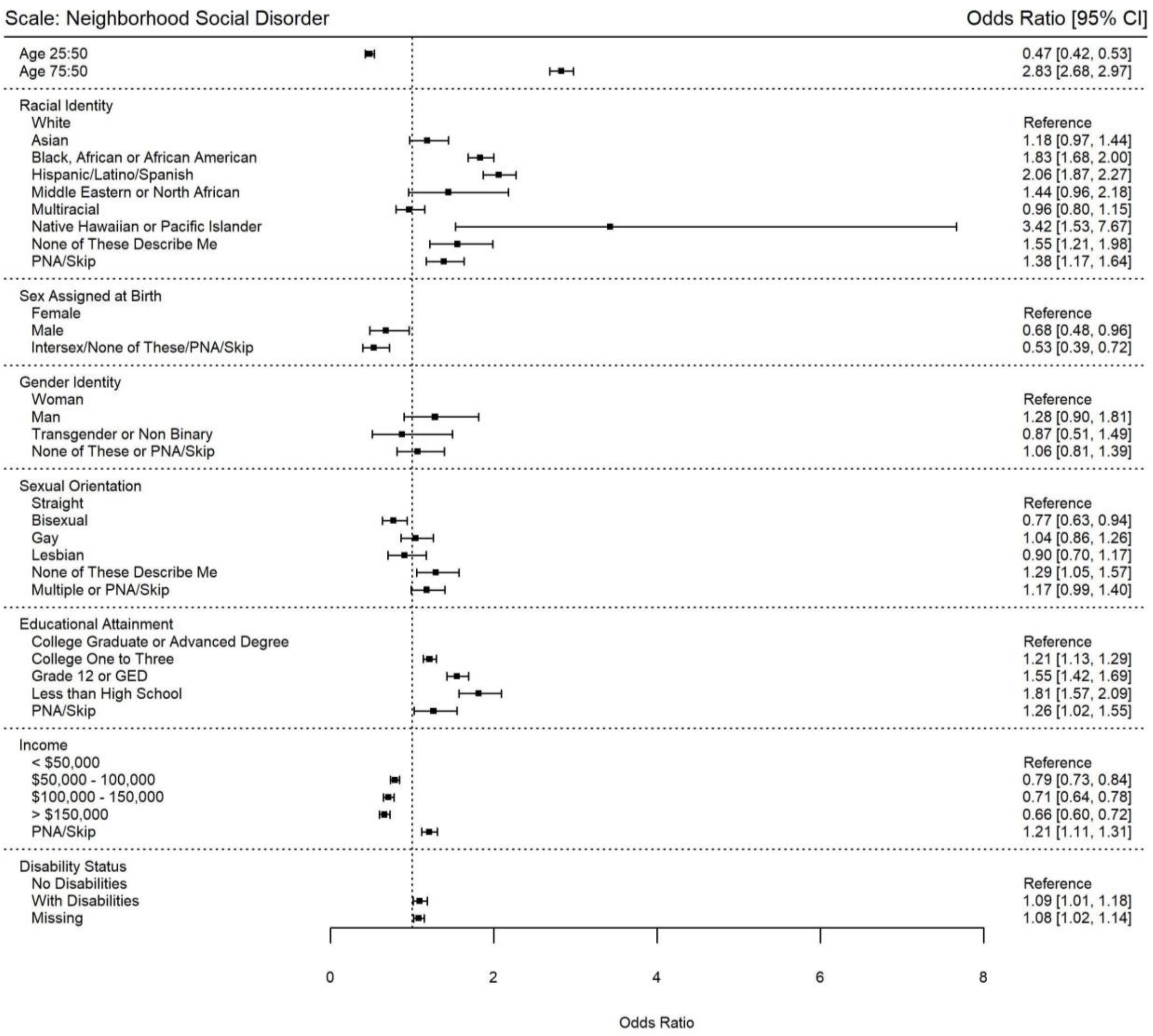

Abbreviations: MENA, Middle Eastern or North African; NHPI, Native Hawaiian or Pacific Islander; PNA, prefer not to answer
Sex at Birth: Sex Assigned at Birth

Supplementary Figure A4: Odds of item non-response or incalculable score for the Everyday Discrimination Scale.

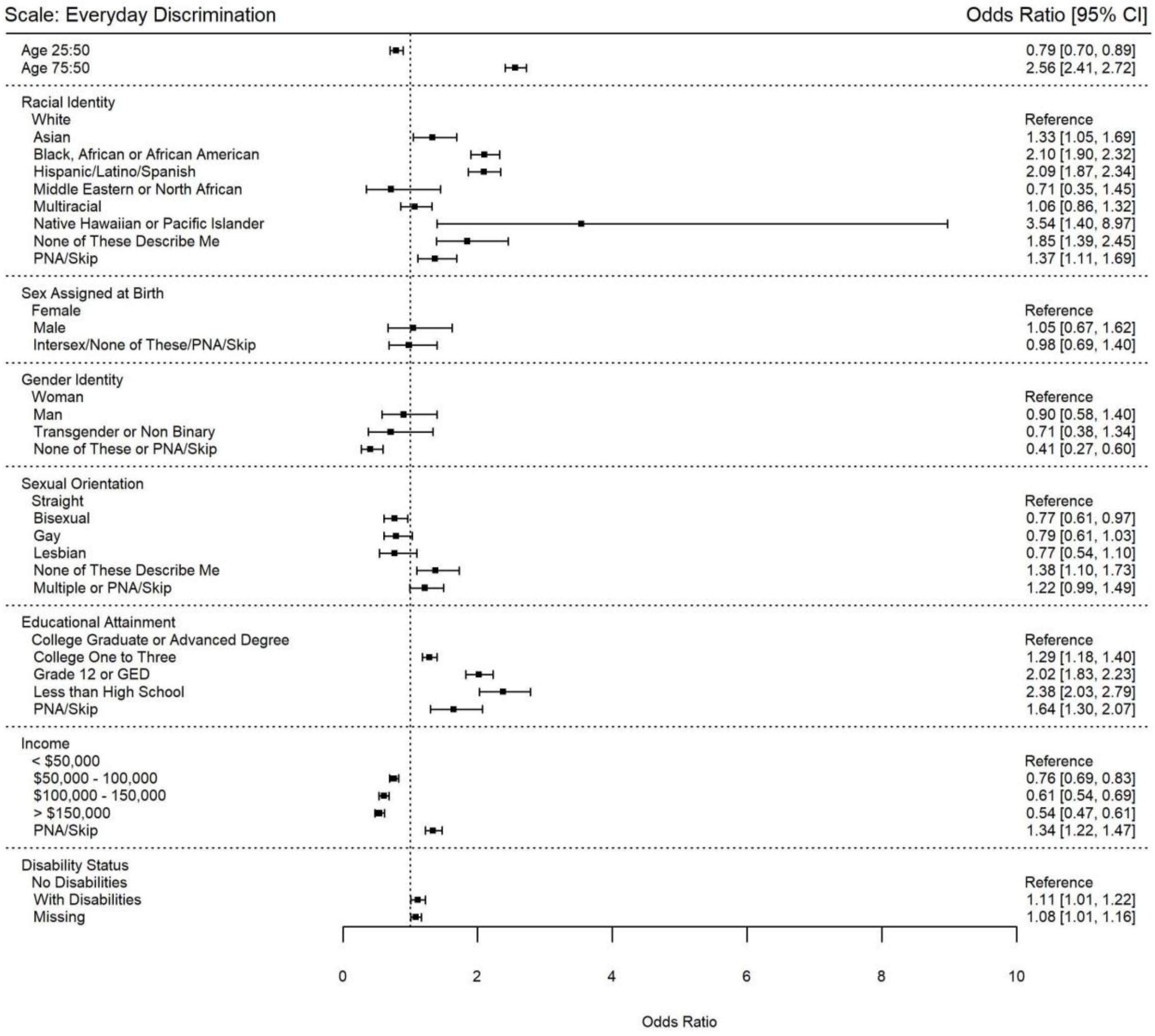

Abbreviations: MENA, Middle Eastern or North African; NHPI, Native Hawaiian or Pacific Islander; PNA, prefer not to answer
Sex at Birth: Sex Assigned at Birth

Supplementary Figure A5: Odds of item non-response or incalculable score for the Discrimination in Medical Settings Scale.

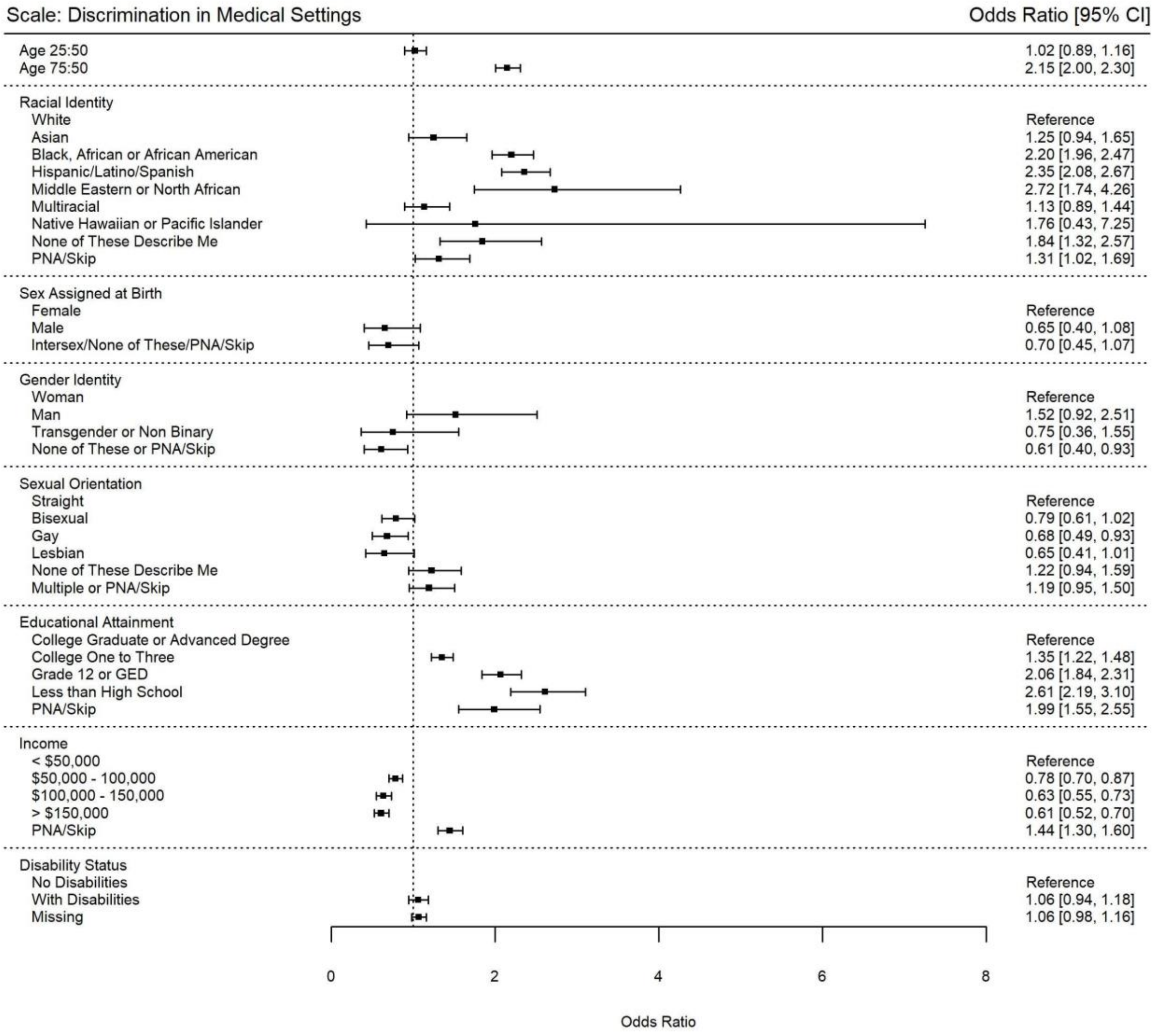

Abbreviations: MENA, Middle Eastern or North African; NHPI, Native Hawaiian or Pacific Islander; PNA, prefer not to answer
Sex at Birth: Sex Assigned at Birth

Supplementary Figure A6: Odds of item non-response or incalculable score for the Social Cohesion Scale.

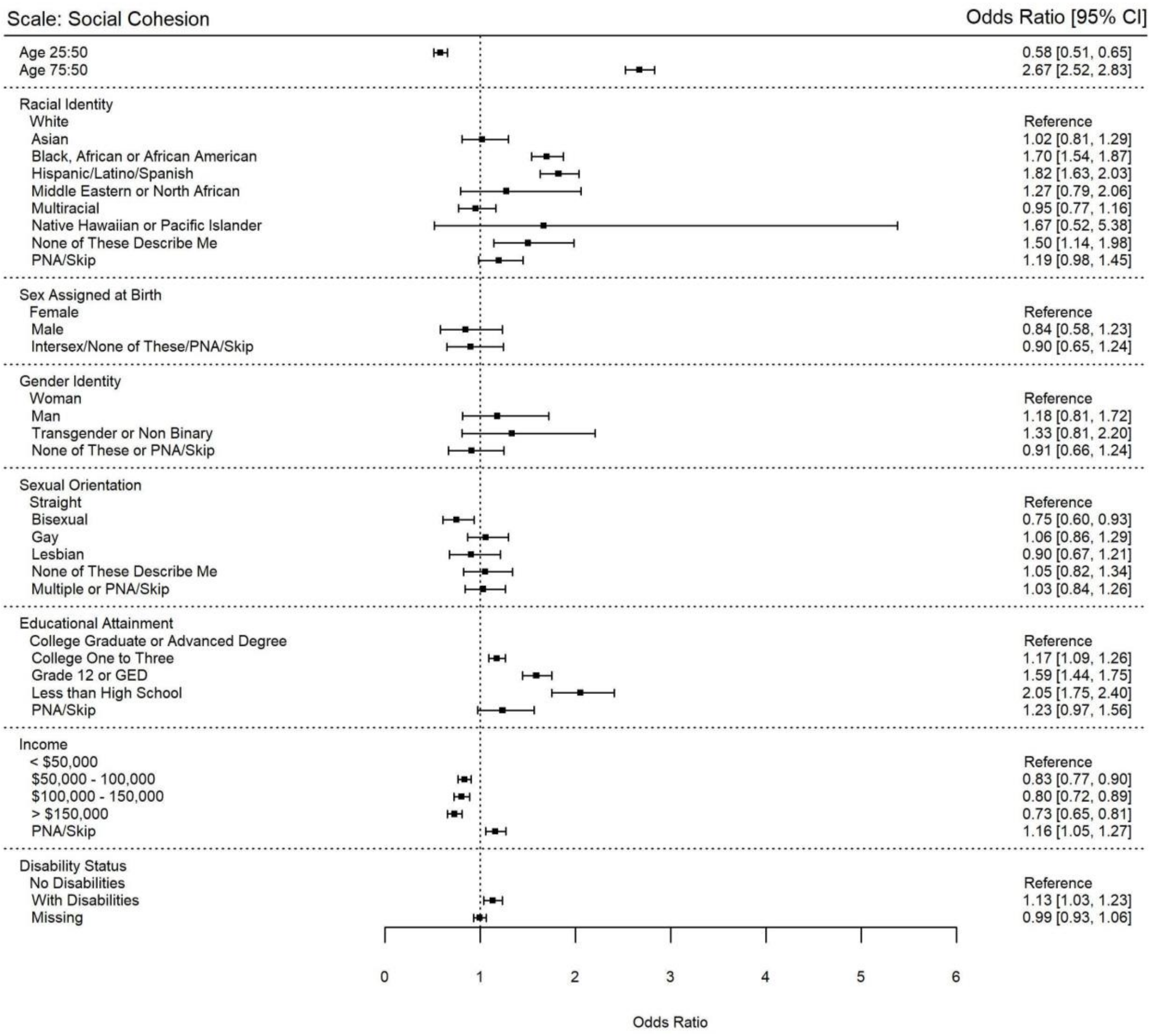

Abbreviations: MENA, Middle Eastern or North African; NHPI, Native Hawaiian or Pacific Islander; PNA, prefer not to answer
Sex at Birth: Sex Assigned at Birth

Supplementary Figure A7: Odds of item non-response or incalculable score for the Perceived Stress Scale.

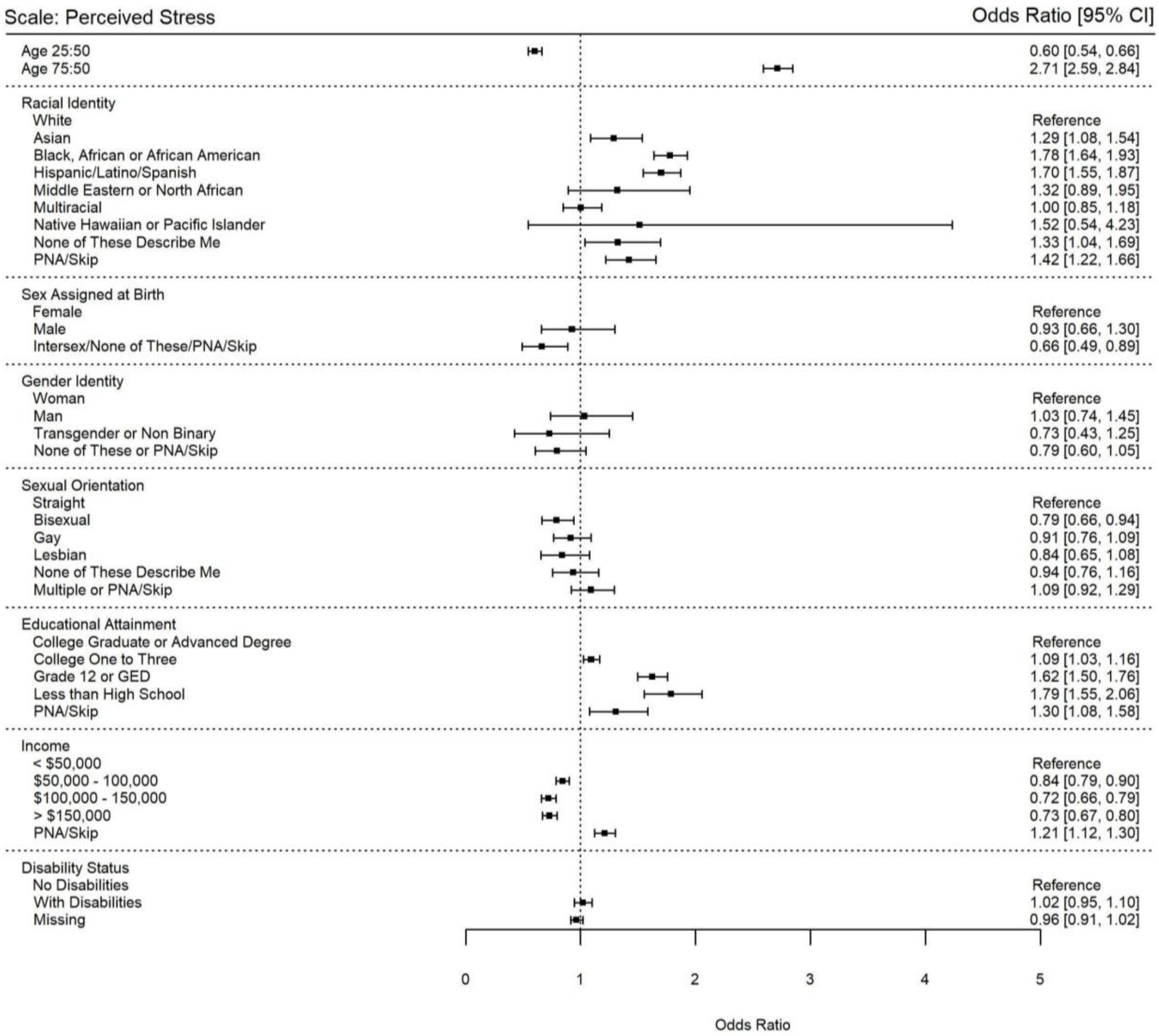

Abbreviations: MENA, Middle Eastern or North African; NHPI, Native Hawaiian or Pacific Islander; PNA, prefer not to answer
Sex at Birth: Sex Assigned at Birth

Supplementary Figure A8: Odds of item non-response or incalculable score for the Total Social Support Scale.

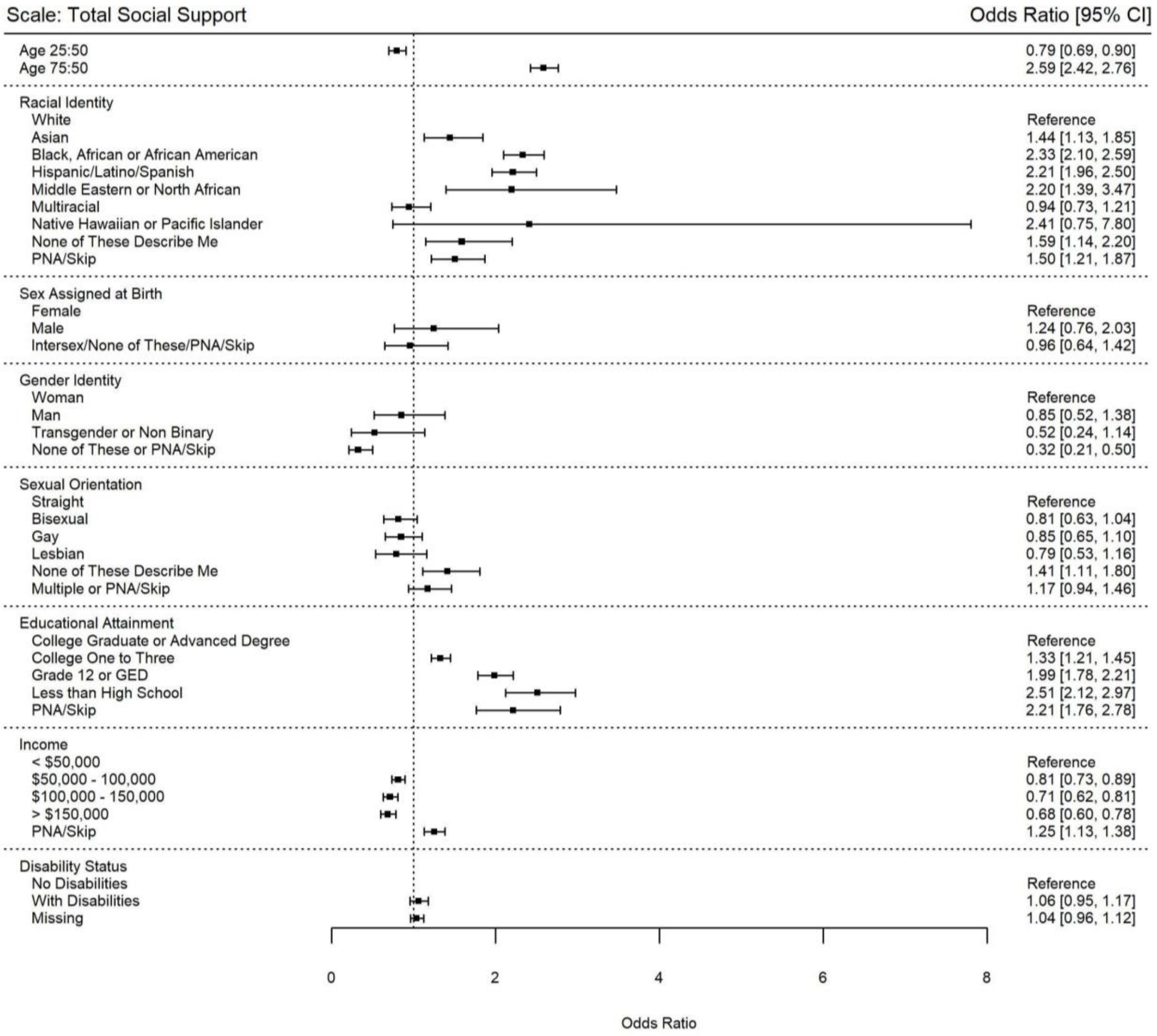

Abbreviations: MENA, Middle Eastern or North African; NHPI, Native Hawaiian or Pacific Islander; PNA, prefer not to answer

Sex at Birth: Sex Assigned at Birth

Supplementary Figure A9: Odds of item non-response or incalculable score for the Instrumental Social Support Scale

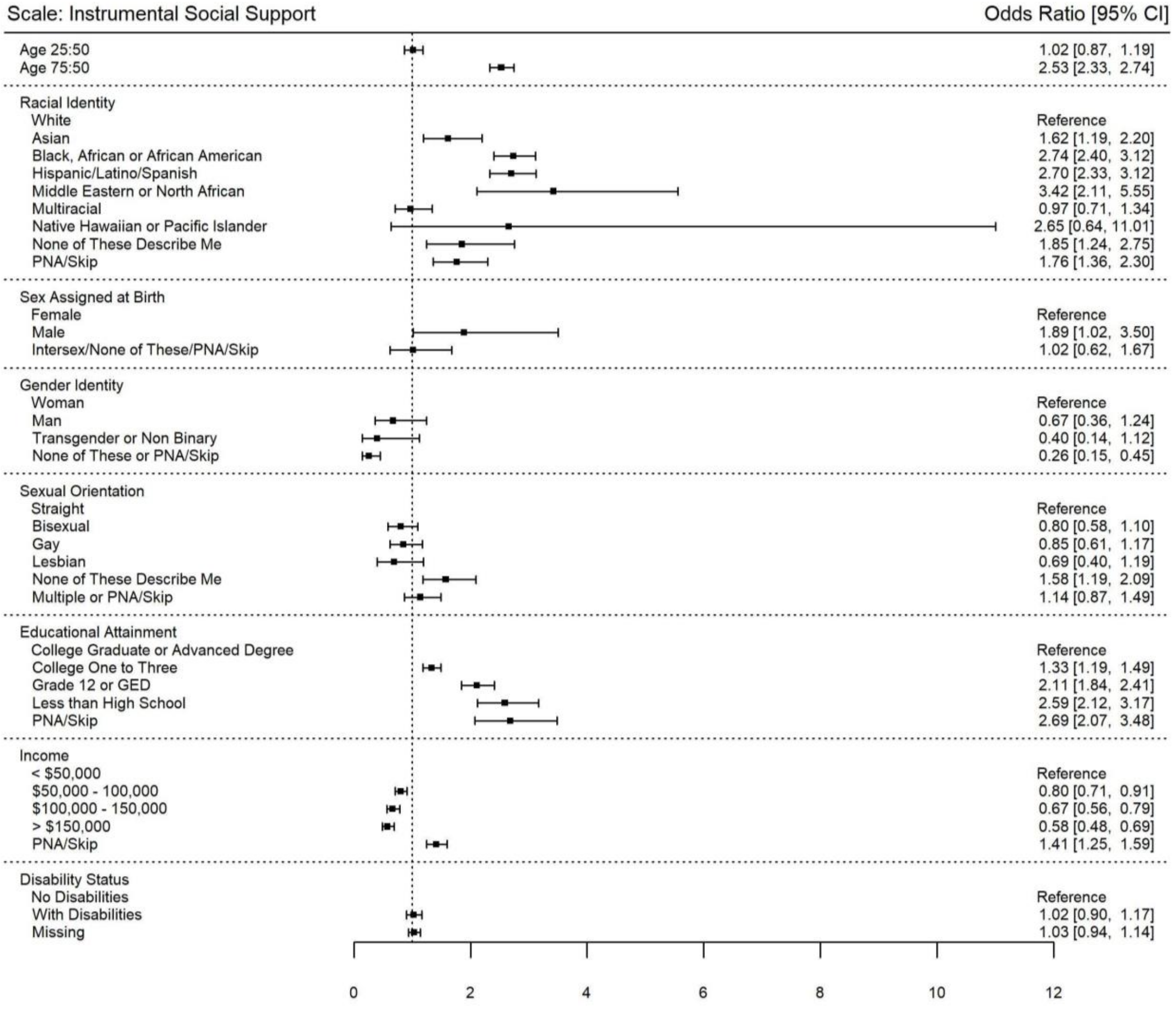

Abbreviations: MENA, Middle Eastern or North African; NHPI, Native Hawaiian or Pacific Islander; PNA, prefer not to answer
Sex at Birth: Sex Assigned at Birth

Supplementary Figure A10: Odds of item non-response or incalculable score for the Emotional Social Support Scale.

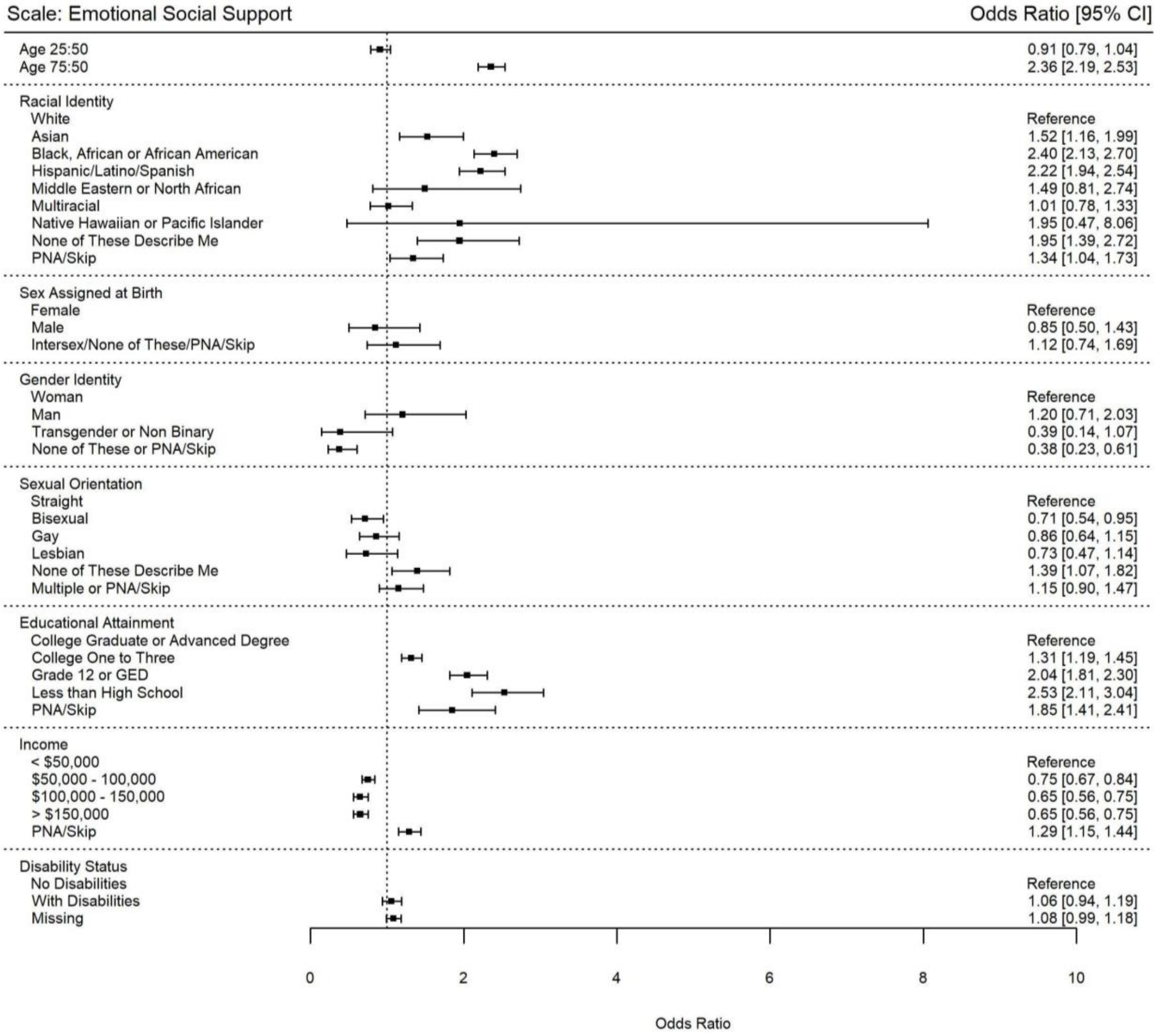

Abbreviations: MENA, Middle Eastern or North African; NHPI, Native Hawaiian or Pacific Islander; PNA, prefer not to answer
Sex at Birth: Sex Assigned at Birth

Supplementary Figure A11: Odds of item non-response or incalculable score for the Daily Spiritual Experiences Scale.

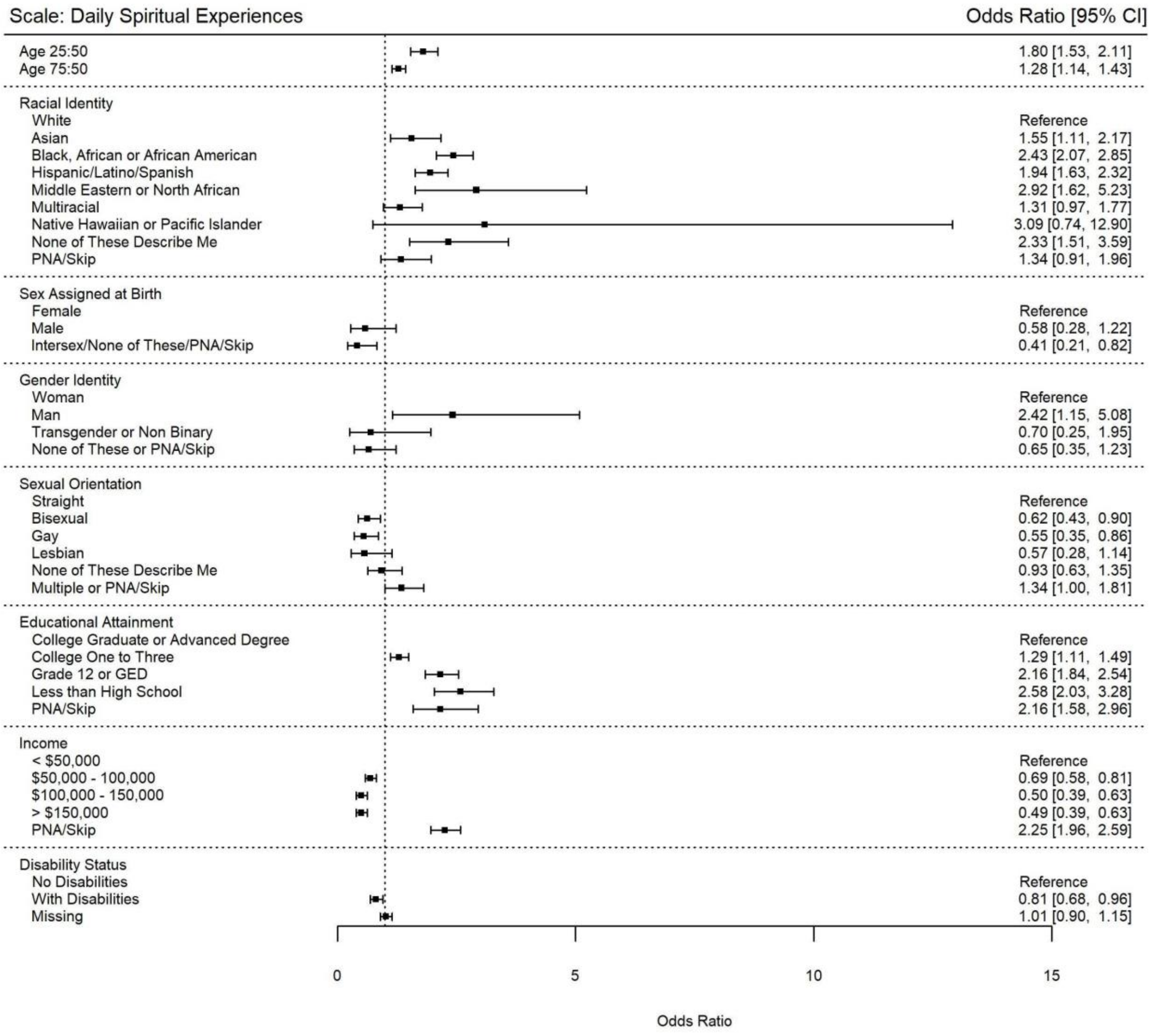

Abbreviations: MENA, Middle Eastern or North African; NHPI, Native Hawaiian or Pacific Islander; PNA, prefer not to answer
Sex at Birth: Sex Assigned at Birth

Supplementary Figure A12: Odds of item non-response or incalculable score for the Housing Instability Scale.

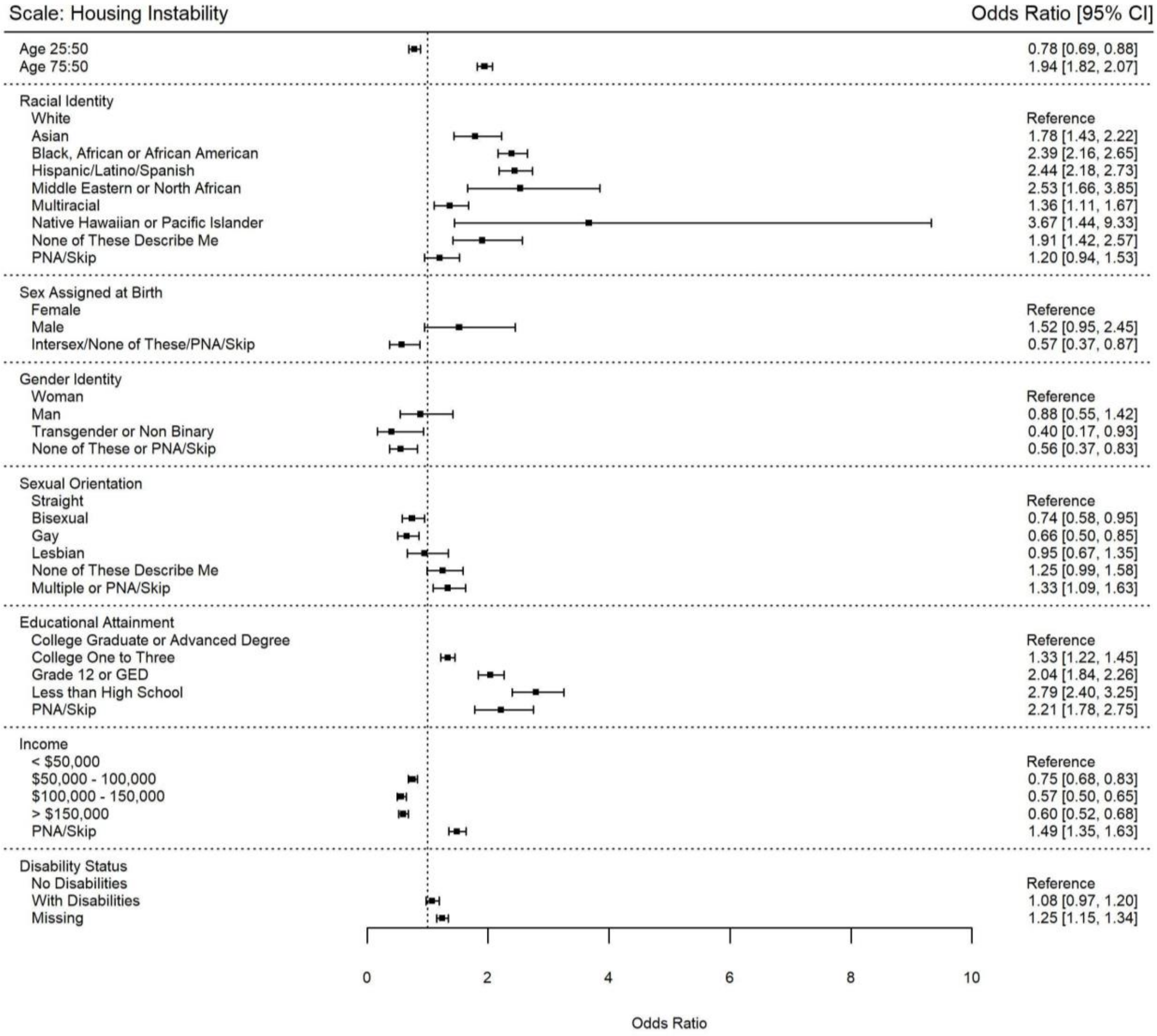

Abbreviations: MENA, Middle Eastern or North African; NHPI, Native Hawaiian or Pacific Islander; PNA, prefer not to answer
Sex at Birth: Sex Assigned at Birth

Supplementary Figure A13: Odds of item non-response or incalculable score for the Food Insecurity Scale.

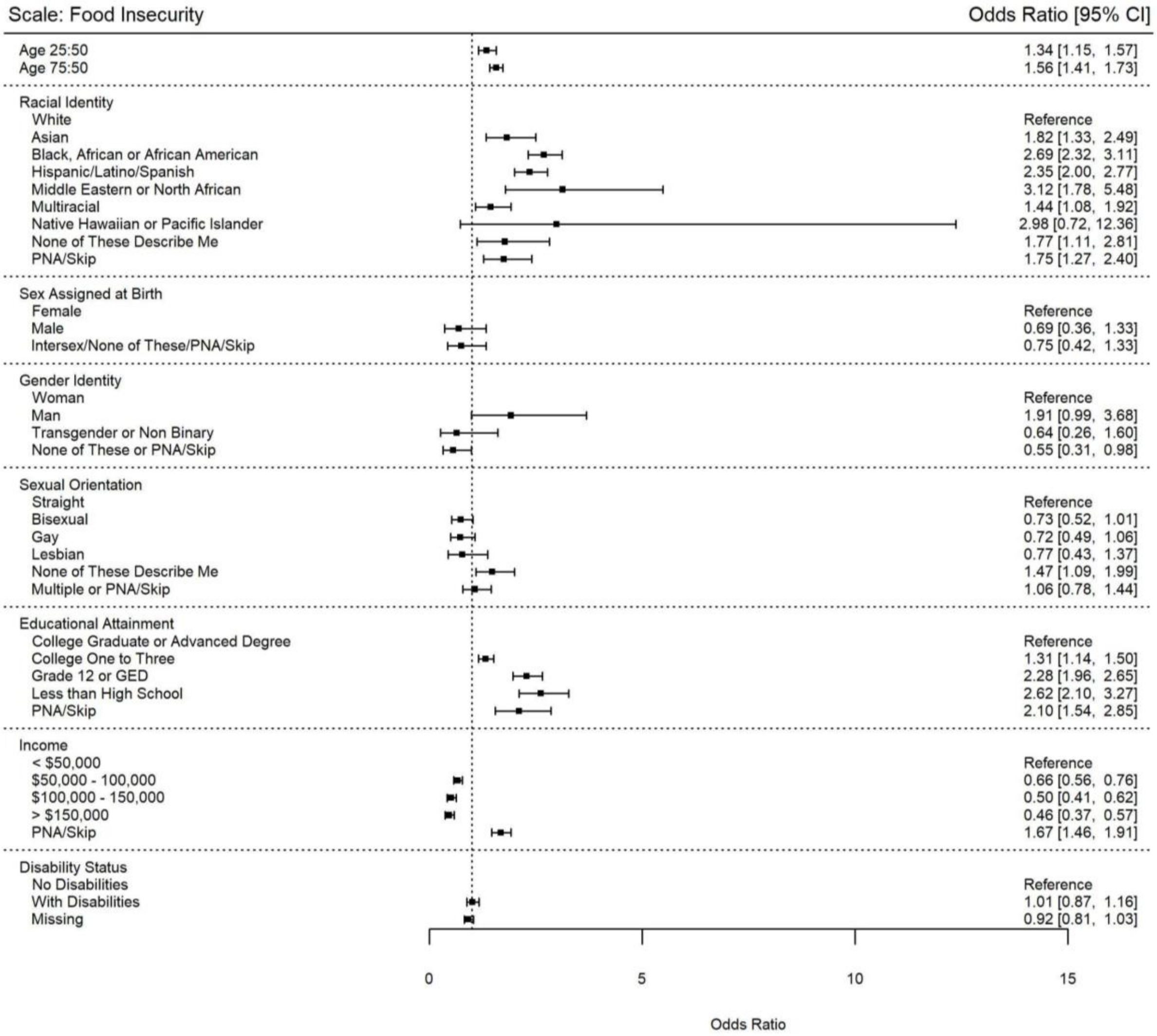

Abbreviations: MENA, Middle Eastern or North African; NHPI, Native Hawaiian or Pacific Islander; PNA, prefer not to answer
Sex at Birth: Sex Assigned at Birth

Supplementary Figure A14: Odds of item non-response or incalculable score for the PANES - Walking and Bicycling Scale.

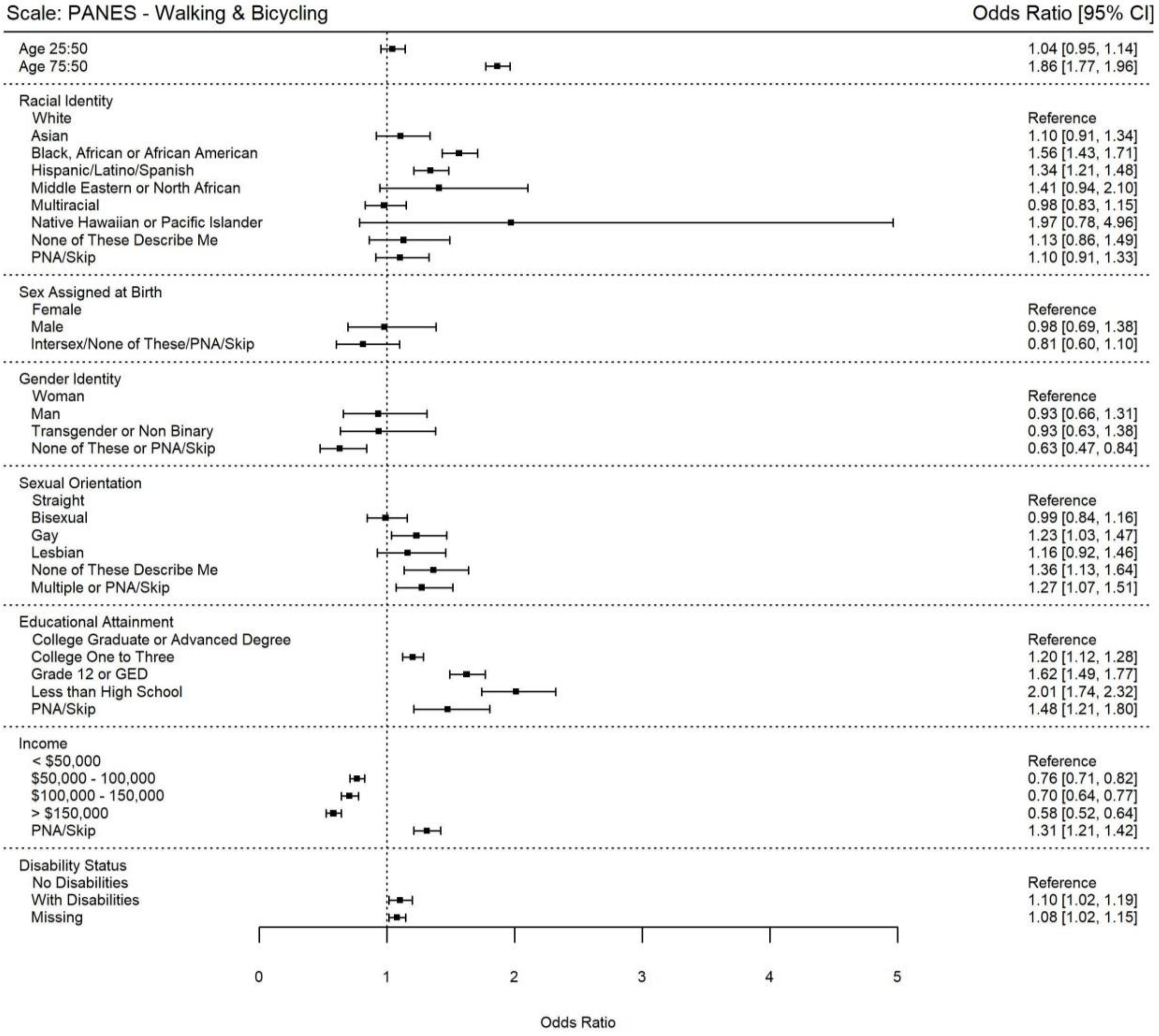

Abbreviations: PANES, Physical Activity and Neighborhood Environment Scale; MENA, Middle Eastern or North African; NHPI, Native Hawaiian or Pacific Islander; PNA, prefer not to answer Sex at Birth: Sex Assigned at Birth

Supplementary Figure A15: Odds of item non-response or incalculable score for the PANES - Crime and Safety Scale.

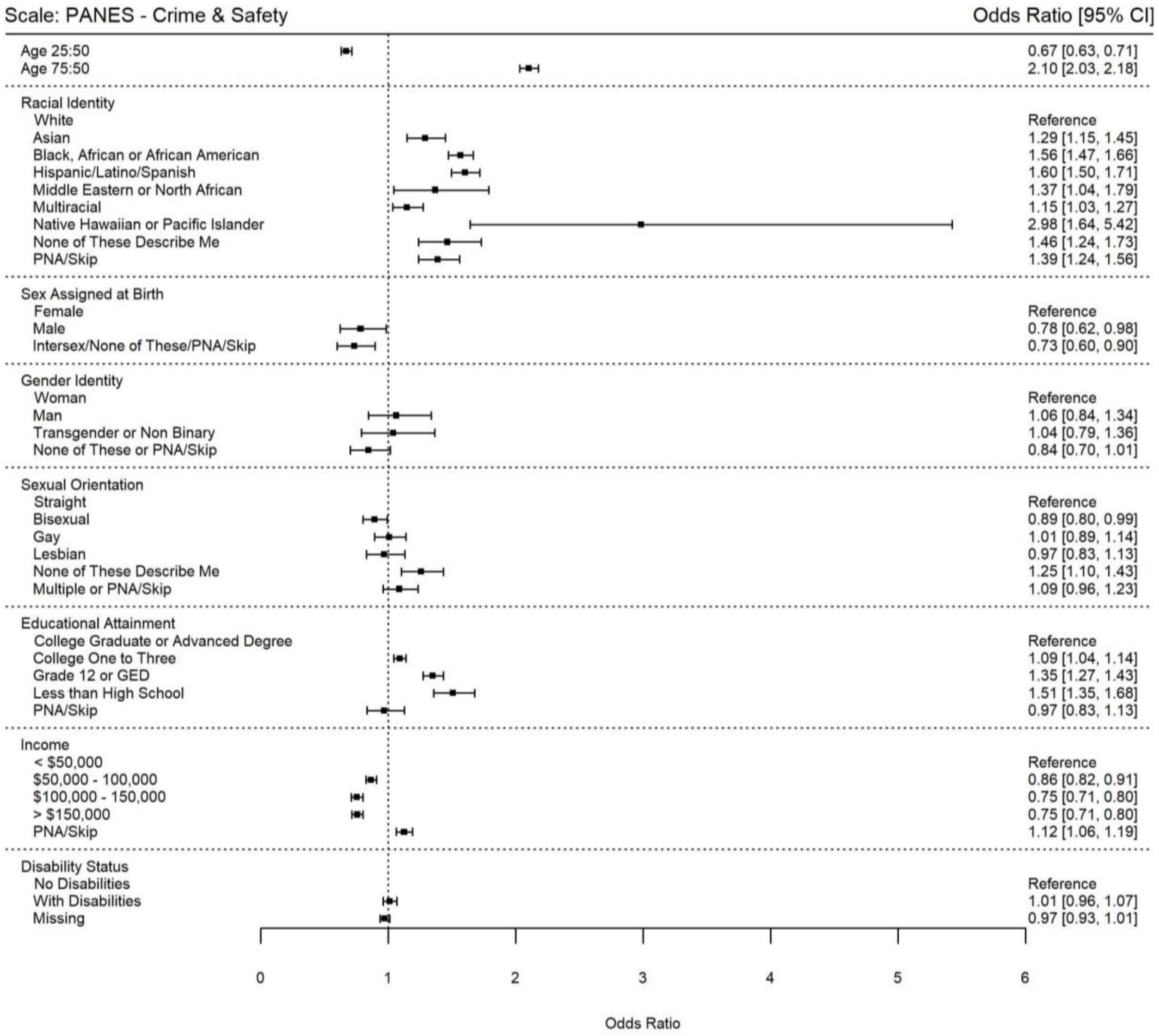

Abbreviations: PANES, Physical Activity and Neighborhood Environment Scale; MENA, Middle Eastern or North African; NHPI, Native Hawaiian or Pacific Islander; PNA, prefer not to answer Sex at Birth: Sex Assigned at Birth

Supplementary Figure A16: Odds of item non-response or incalculable score for the Housing Quality Scale.

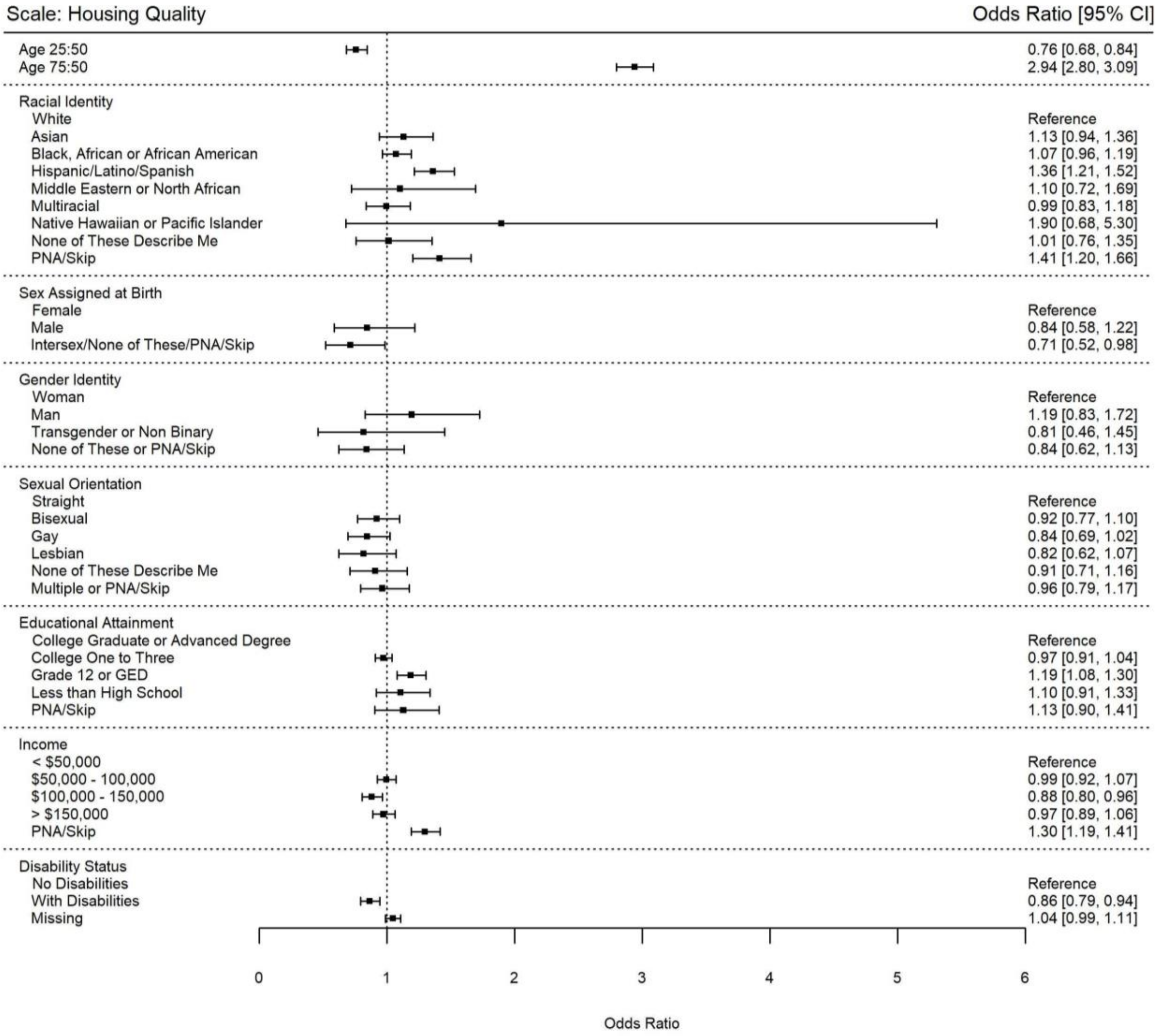

Abbreviations: MENA, Middle Eastern or North African; NHPI, Native Hawaiian or Pacific Islander; PNA, prefer not to answer
Sex at Birth: Sex Assigned at Birth

Supplementary Figure A17: Odds of item non-response or incalculable score for the Religious Attendance Scale.

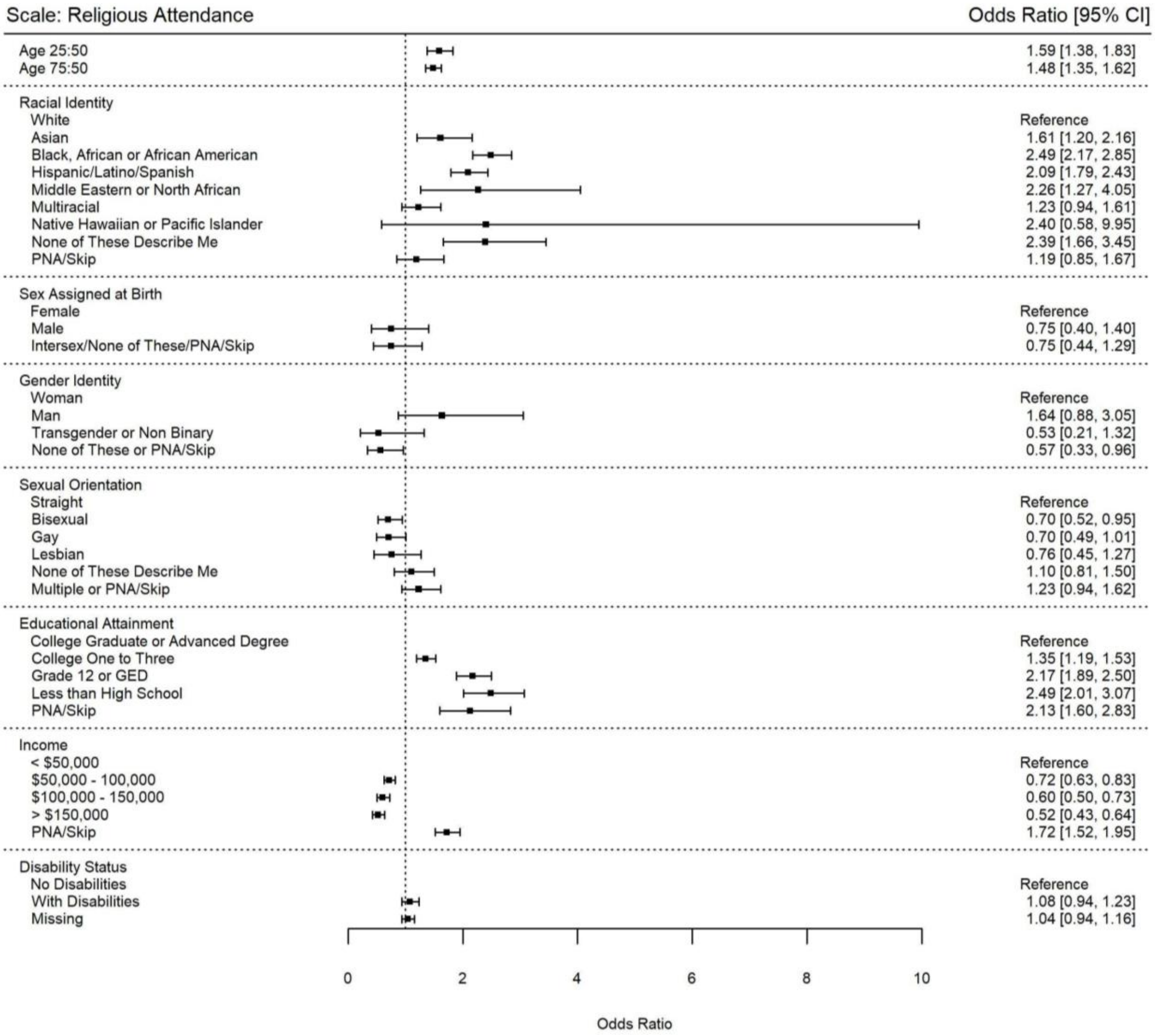

Abbreviations: MENA, Middle Eastern or North African; NHPI, Native Hawaiian or Pacific Islander; PNA, prefer not to answer

Sex at Birth: Sex Assigned at Birth

Supplementary Note: Acknowledgement List of Principal Investigators

Past and Present *All of Us* Research Program Principal Investigators

Brian Ahmedani[1]; Christine D Cole Johnson[1]; Habib Ahsan[2]; Hoda Anton-Culver[4]; Eric Topol[5]; Katie Baca-Motes[5]; Julia Moore-Vogel[5]; Praduman Jain[6]; Mark Begale[6]; Neeta Jain[6]; David Klein, MBA[6]; Scott Sutherland[6]; Bruce Korf[7]; Beth Lewis[7]; Ali G Gharavi[8]; George Hripcsak[8]; Eric Boerwinkle[9]; Scott Joseph Hebbring[10]; Elizabeth Burnside[11]; Dorothy Farrar-Edwards[11]; Amy Taylor[12]; Liliana Lombardi Desa[13]; Steve Thibodeau[14]; Mine Cicek[14]; Eric Schlueter[15]; Beverly Wilson Holmes[15]; Martha Daviglus[16]; Paul Harris[+17]; Consuelo Wilkins[17]; Dan Roden[17]; Kim Doheny[18]; Evan Eichler[19]; Gail Jarvik[19]; Gretchen Funk[20]; Anthony Philippakis[21]; Heidi Rehm[21]; Stacey Gabriel[21]; Richard Gibbs[22]; Edgar M Gil Rico[23]; David Glazer[24]; Jessica Burke[25]; Philip Greenland[26]; Elizabeth Shenkman[27]; William R Hogan[27]; Priscilla Igho-Pemu[28]; Elizabeth W Karlson[29]; Jordan Smoller[29]; Shawn N Murphy[29]; Cheryl R. Clark[51]; Margaret Elizabeth Ross[30]; Rainu Kaushal[30]; Eboni Winford[31]; Vik Kheterpal[32]; Francisco A Moreno[33]; Cheryl Thomas[34]; Mitchell Lunn[35]; Juno Obedin-Maliver[35]; Oscar Marroquin[36]; Shyam Visweswaran[36]; Steven Reis[36]; Patrick McGovern[37]; Gregory Talavera[38]; George T O'Connor[39]; Lucila Ohno-Machado[41]; Fornessa Randal[43]; Andreas A Theodorou[44]; Eric Reiman[44]; Mercedita Roxas-Murray[45]; Louisa Stark[46]; Ronnie Tepp[47]; Alicia Zhou[48]; Scott Topper[48]; Rhonda Trousdale[49]; Phil Tsao[50]; Scott T Weiss[51]; Jeffrey Whittle[53]; Stephan Zuchner[55]; Olveen Carrasquillo[55]; Megan Lewis[57]; Jen Uhrig[57]; May Okihiro[58]; Maria Argos[16]; Brisa Aschebook-Kilfoy[16]; Laura Bartlett[54]; Roberta Carlin[59]; Elizabeth Cohn[60]; Vivian Colon-Lopez[61]; Karl Cooper[59]; Linda Cottler[62]; Errol Crook[63]; Elizabeth Culler[64]; Charles Drum[59]; Milton Eder[62]; Mark Edmunds[52]; Rachel Everhart[65]; Adolph Falcon[23]; Becky Fein[66]; Zeno Frano[53]; Michael Garrett[67]; Sandra Halverson[68]; Eileen Handberg[27]; Joyce Ho[26]; Laura Horne[66]; Rosario Isasi[55]; Jessica Isom[69]; Jessica Jarmin[70]; Megan Jula[71]; Royan Kamyar[72]; Frida Kleiman[60]; Isaac Kohane[73]; Babbette Lamarca[67]; Brendan Lee[22]; Niall Lennon[21]; Dessie Levy[74]; Todd Mahr[75]; Emily Makahi[58]; Vivienne Marshall[76];Elizabeth Mayer-Davis[77]; Jacob McCauley[55]; Jeffrey McKinney[78]; David McPherson[9]; Robert Meller[28]; Jose Melo[61]; David Ming-Hung Lin[79]; Michael Minor[74]; Evan Muse[5]; Kapil Parakh[80]; Cathryn Peltz-Rauchman[1]; Linda Rose Perez Laras[81]; Subhara Raveendran[82]; Gail Reilly[31]; Jody Reilly[83]; Nelida Rivera[81]; Laura Rosales[22]; Tracie Rosser[56]; Linda Salgin[38]; Sherilyn Sawyer[84]; William Simonson[85]; Amy Sitapati[41]; Cynthia So-Armah[69]; Gene Stegeman[86]; Christin Suver[87]; Michael Taitel[42]; Kyla Taylor[31]; Daniel Hernandez Tinoco[31]; Scott Topper[48]; Rhonda Trousdale[49]; Jason Vassy[84]; Jamie Walz[78]; Preston Watkins[88]; Blaker Wilkerson[89]; Katrina Yamazaki[12]; Melissa Basford[17]; Amrylis Silva Boschetti[41]; Suchitra Chandrasekaran[56]; Kim Enard[90]; Yuri Fresko[83]; Richard Grucza[90]; Robert Kelley[56]; Kathleen Keogh[13]; Cora Elizabeth Lewis[7]; Christopher Lough[91]; Ted Malmstrom[90]; David Ming-Hung Lin[79]; Paul Nemeskal[69]; Matt Pagel[56]; Jeffrey Scherrer[90]; Sanjay Shukla[10]; Debra Smith[92]; Bryce Turner[93]; Miriam Vos[56]

Note

This is the list of individuals who were Principal Investigators or equivalent with the *All of Us* Research Program during the period that this paper was in development, March 1, 2022 - June 1, 2023.

Legend

+ Principal Investigator/Lead Author for the *All of Us* Research Program protocol (paul.a.harris@vumc.org)

Affiliations

1. Henry Ford Health System
2. University of Chicago Medical Center
3. Jackson-Hinds Comprehensive Health Center
4. University of California, Irvine
5. Scripps Research Translational Institute
6. Vibrent Health
7. University of Alabama at Birmingham
8. Columbia University
9. University of Texas Health Science Center at Houston
10. Marshfield Clinic Research Institute
11. University of Wisconsin at Madison
12. Community Health Center, Inc.
13. Sun River Health
14. Mayo Clinic and Foundation, Rochester
15. Cooperative Health
16. University of Illinois at Chicago
17. Vanderbilt University Medical Center
18. Johns Hopkins University School of Medicine
19. University of Washington
20. FiftyForward
21. Broad Institute
22. Baylor University
23. National Alliance for Hispanic Health
24. Verily Life Sciences

25. MITRE Corporation
26. Northwestern University
27. University of Florida
28. Morehouse School of Medicine, Atlanta
29. Partners Health Care
30. Cornell University, Weill Medical College
31. Cherokee Health Systems
32. CareEvolution, Inc.
33. University of Arizona, Tucson
34. Delta Research and Educational Foundation
35. Stanford University
36. University of Pittsburgh
37. Wondros
38. San Ysidro Health Center
39. Boston Medical Center
40. VA *All of Us* Coordinating Center, Boston
41. University of California, San Diego
42. Walgreen Co.
43. Asian Health Coalition
44. Banner Health
45. Montage Marketing Group
46. University of Utah
47. HCM Strategists
48. Color Genomics, Inc.
49. NYC Health + Hospitals
50. VA AoU Coordinating Center - Palo Alto
51. Brigham and Women's Hospital
52. San Diego Blood Bank
53. Medical College of Wisconsin

54. National Library of Medicine (NLM)
55. University of Miami School of Medicine
56. Emory University
57. Research Triangle Institute
58. Waianae Coast CHC
59. American Association of Health and Disability
60. Hunter College
61. University of Puerto Rico Comprehensive Cancer Center
62. CTSA Community Engagement Programs
63. University of South Alabama
64. TPC: Blood Assurance
65. TPC: Denver Health
66. TPC: Active Minds
67. University of Mississippi Medical Center
68. TPC: DLH Corp
69. Mass General Hospital
70. Tactis
71. TPC: Mary’s Center
72. TPC: Owaves
73. Harvard Medical School
74. National Baptist Convention
75. Gundersen Health System
76. South Texas Blood and Tissue Center
77. University of North Carolina at Chapel Hill
78. Sensis
79. TPC: Bloodworks Northwest
80. TPC: Fitbit
81. COSSMA
82. Patients Like Me

83. Quest Diagnostics Incorporated
84. VA AoU Coordinating Center
85. Cascade Regional BLood Services
86. ExamOne
87. Sage Bionetworks
88. WebMD Health Corp
89. Blue Cross Blue Shield
90. Saint Louis University
91. TPC: LifeSouth
92. TPC: SunCoast Blood Center
93. University of Southern California
